# Claudin18.2-specific CAR T cells in gastrointestinal cancers: phase 1 trial interim results

**DOI:** 10.1038/s41591-022-01800-8

**Published:** 2022-05-09

**Authors:** Changsong Qi, Jifang Gong, Jian Li, Dan Liu, Yanru Qin, Sai Ge, Miao Zhang, Zhi Peng, Jun Zhou, Yanshuo Cao, Xiaotian Zhang, Zhihao Lu, Ming Lu, Jiajia Yuan, Zhenghang Wang, Yakun Wang, Xiaohui Peng, Huiping Gao, Zhen Liu, Huamao Wang, Daijing Yuan, Jun Xiao, Hong Ma, Wei Wang, Zonghai Li, Lin Shen

**Affiliations:** 1 Department of Gastrointestinal Oncology, Key Laboratory of Carcinogenesis and Translational Research (Ministry of Education/Beijing), Peking University Cancer Hospital and Institute, Beijing, China; 2 Key Laboratory of Carcinogenesis and Translational Research (Ministry of Education/Beijing), Early Drug Development Center, Peking University Cancer Hospital and Institute, Beijing, China; 3 Department of Oncology, The First Affiliated Hospital of Zhengzhou University, Zhengzhou, China; 4 CARsgen Therapeutics Co., Ltd., Shanghai, China

**Keywords:** Cancer immunotherapy, Gastric cancer, Gastrointestinal cancer, T cells, Phase I trials

## Abstract

Despite success in hematologic malignancies, the treatment landscape of chimeric antigen receptor (CAR) T cell therapy for solid tumors remains limited. Claudin18.2 (CLDN18.2)-redirected CAR T cells showed promising efficacy against gastric cancer (GC) in a preclinical study. Here we report the interim analysis results of an ongoing, open-label, single-arm, phase 1 clinical trial of CLDN18.2-targeted CAR T cells (CT041) in patients with previously treated, CLDN18.2-positive digestive system cancers (NCT03874897). The primary objective was safety after CT041 infusion; secondary objectives included CT041 efficacy, pharmacokinetics and immunogenicity. We treated 37 patients with one of three CT041 doses: 2.5 × 10^8^, 3.75 × 10^8^ or 5.0 × 10^8^ cells. All patients experienced a grade 3 or higher hematologic toxicity. Grade 1 or 2 cytokine release syndrome (CRS) occurred in 94.6% of patients. No grade 3 or higher CRS or neurotoxicities, treatment-related deaths or dose-limiting toxicities were reported. The overall response rate (ORR) and disease control rate (DCR) reached 48.6% and 73.0%, respectively. The 6-month duration of response rate was 44.8%. In patients with GC, the ORR and DCR reached 57.1% and 75.0%, respectively, and the 6-month overall survival rate was 81.2%. These initial results suggest that CT041 has promising efficacy with an acceptable safety profile in patients with heavily pretreated, CLDN18.2-positive digestive system cancers, particularly in those with GC.

## Abstract (web summary)

In an interim analysis of a phase 1 trial, CAR T cells specific for Claudin18.2, a tight junction protein isoform highly expressed on gastrointestinal tract tumors, were well-tolerated and exhibited promising clinical activity in patients with gastrointestinal cancers.

## Main

Although CAR T cell therapy has displayed remarkable clinical efficacy in various types of hematologic malignancies^1–4^, its efficacy in solid tumors is still very limited^5–7^. CLDN18.2 is the gastric-specific isoform of the tight junction protein of CLDN18. It is highly expressed in multiple cancers, especially in cancers of the digestive system, making it a potential target for anti-tumor therapy^8,9^. The monoclonal antibody zolbetuximab is the first CLDN18.2-targeted treatment under clinical development. As a monotherapy, zolbetuximab reported an ORR of 9% in CLDN18.2-positive patients with recurrent or refractory advanced gastric cancer/gastroesophageal junction (GC/GEJ) cancer who had received at least one prior line of therapy^10^. Zolbetuximab combined with epirubicin, oxaliplatin and capecitabine (EOX) achieved an ORR of 39.0% versus 25.0% (*P* = 0.034), and a duration of response (DOR) of 32.6 weeks versus 21.7 weeks (*P* = 0.023) when compared to EOX alone in CLDN18.2-positive patients with advanced GC/GEJ cancer in the first-line setting^11^. As the mechanism of action between monoclonal antibodies and CAR T cells is known to be quite different, it is necessary to explore the anti-tumor activities of CLDN18.2 CAR-T in gastrointestinal (GI) cancer.

**Extended Data Fig. 1:**
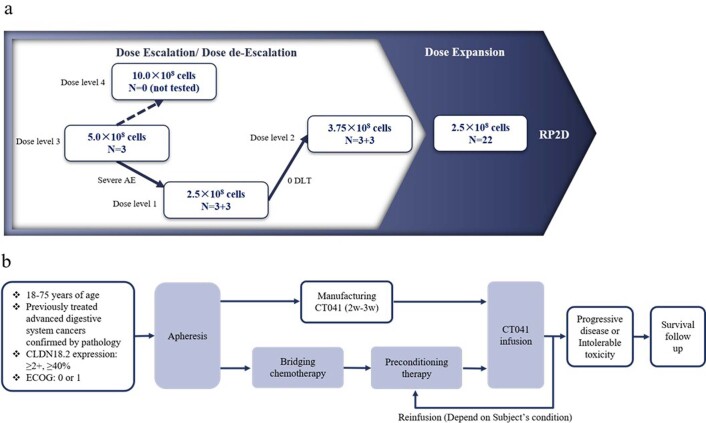
Study flow diagram and procedure schema. **a**, Two-phase study design: a dose escalation/de-escalation phase and a dose-expansion phase to determine the recommended phase II dose (RP2D). **b**, Schema of study procedure. DLT, dose-limiting toxicity; ECOG, Eastern Cooperative Oncology Group performance status.

CT041 contains genetically engineered autologous T cells that express the CLDN18.2-targeted CAR. The CAR structure consists of a humanized anti-CLDN18.2 single-chain variable fragment, a CD8α hinge region, a CD28 co-stimulatory domain and a CD3ζ signaling domain (Supplementary Fig. 1). Our previous preclinical data indicated that CT041 had antigen-specific anti-tumor effects on GC, leading to significant tumor regression without weight loss in immunodeficient mice^12^. GC has a poor prognosis with limited treatment options, including first-line monotherapies or combinations of oxaliplatin, fluoropyrimidine, irinotecan, paclitaxel, anti-PD-1 antibody, docetaxel and carboplatin with or without trastuzumab depending on HER2 expression status, as well as ramucirumab (VEGFR-2 monoclonal antibody) plus paclitaxel as second-line treatment^13^. Similarly, limited treatments exist for metastatic pancreatic cancer (PC), including gemcitabine with or without nab-paclitaxel, FOLFIRINOX and nanoliposomal irinotecan with fluorouracil^14^. A previous single-center clinical trial (NCT03159819) indicated that CT041 was well-tolerated and could be a promising therapy for patients with gastric and pancreatic cancers^15^. Therefore, we initiated an open-label, multicenter, single-arm, dose-escalation/de-escalation and dose-expansion phase 1 trial to investigate the safety, efficacy, pharmacokinetics and immunogenicity of CT041 in patients with cancers of the digestive system (Extended Data Fig. 1). Here we report the outcomes from a non-prespecified interim analysis of this ongoing trial (NCT03874897).

## Results

### Study design and baseline characteristics of patients

The trial recruited adult patients with advanced digestive system cancers whose tumor tissues expressed CLDN18.2 as confirmed by immunohistochemistry (IHC), who had Eastern Cooperative Oncology Group (ECOG) performance status scores of 0 or 1 and who had been treated with at least one line of standard systemic therapy. Patients were excluded from the study if they had a target lesion larger than 4 cm at the time of apheresis; active infection including, but not limited to, hepatitis B virus (HBV), hepatitis C virus (HCV) and tuberculosis; brain metastases; unstable or active gastric ulcer or gastroenterological bleeding; central or widespread metastatic lung lesion(s); or massive liver metastases. A complete list of eligibility criteria can be found in the Methods.

The trial consists of a modified ‘3 + 3’ pattern dose-escalation/de-escalation phase and a dose-expansion phase. In the dose-escalation/de-escalation phase, patients with CLDN18.2-positive tumors received one of three CT041 dose levels: 2.5 × 10^8^, 3.75 × 10^8^ or 5.0 × 10^8^ cells. Based on discussions with the Data Safety Monitoring Committee (DSMC), the 2.5 ×10^8^ CAR T cell dose was recommended for the dose-expansion phase (Extended Data Fig. 1).

**Extended Data Fig. 2:**
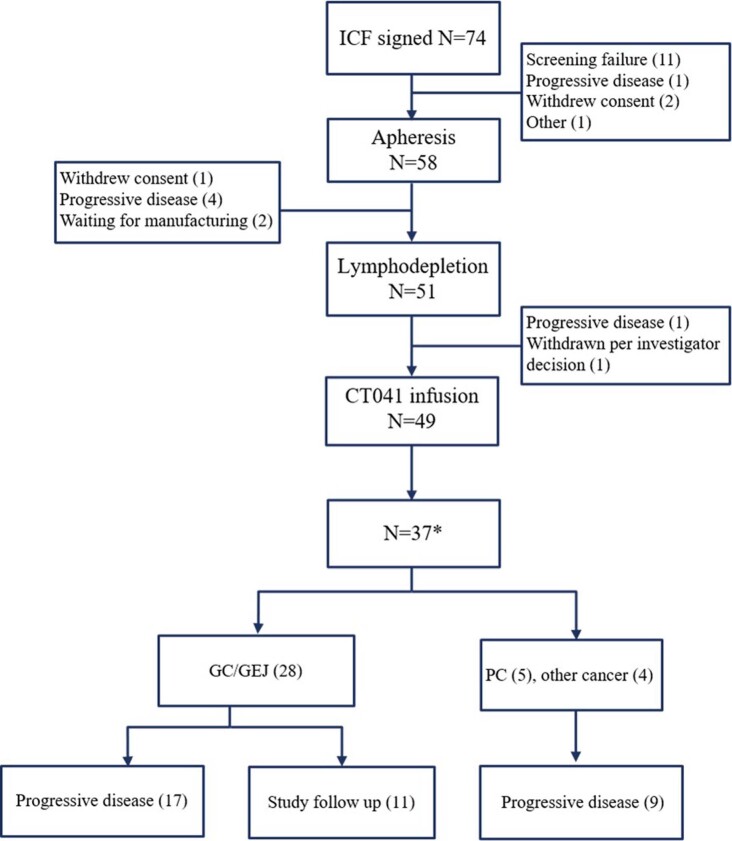
CONSORT Diagram. GC/GEJ, gastric/gastroesophageal junction cancer; PC, pancreatic cancer. * Data are from the first 37 patients who were treated with CT041 and completed at least 12 weeks of follow-up as of the data cutoff date, 08 April 2021.

From 26 March 2019 through 8 April 2021, 74 participants signed informed consent; 59 patients were apheresed; and 49 patients were infused. The first 37 patients were included in this interim analysis, including 28 with GC/GEJ cancer, five with PC and four with other digestive system tumor types (Table 1 and Extended Data Table 1). These patients received CT041 infusion and had completed at least 12 weeks of safety, efficacy and pharmacokinetics assessments after the first infusion (Table 1, Extended Data Table 1 and Extended Data Fig. 2). The median age was 53.0 (25–74) years, and 64.9% (24/37) of patients were men. All enrolled patients were diagnosed with metastatic disease, and 50% of them had at least three organ sites involved. Among the 28 patients with GC/GEJ cancer, 25 (89.3%) had received at least two lines of therapy or at least a triple combination of fluoropyrimidine, oxaliplatin and paclitaxel, and 12 (42.9%) had received prior anti-PD-1/PD-L1 antibody therapy. No HER2-positive patients were enrolled in this study. The cell doses of 2.5 × 10^8^, 3.75 × 10^8^ and 5.0 × 10^8^ were administered to 28, six and three patients, respectively, with a median follow-up of 8.5 months (range, 2.9–19.4 months) (Supplementary Table 1) since apheresis. As of the data cutoff date (8 April 2021) for this interim analysis, patient enrollment continues for the study, and the results will be reported when available.

**Table 1:** Baseline characteristics of all patients and patients with GC who received CT041

All patient characteristics	Dose-escalation/de-escalation *n* = 18	Dose-expansion *n* = 22	Total *n* = 37
Median age (range), years	56.0 (38–74)	43.5 (25–73)	53.0 (25–74)
Male sex, *n* (%)	11 (73.3)	13 (59.1)	24 (64.9)
Disease type, *n* (%)			
GC/GEJ	12 (80)	16 (72.7)	28 (75.7)
PC	2 (13.3)	3 (13.6)	5 (13.5)
Other GI	1 (6.7)	3 (13.6)	4 (10.8)
Median time since metastases (range), years	1.20 (0.1–1.9)	1.00 (0.2–3.2)	1.00 (0.1–3.2)
Peritoneal metastases, n (%)	11 (73.3)	12 (54.5)	23 (62.2)
ECOG score, *n* (%)			
0	2 (13.3)	0	2 (5.4)
1	13 (86.7)	22 (100)	35 (94.6)
Bridging therapy, *n* (%)	12 (80.0)	16 (72.7)	28 (75.7)
CLDN18.2 expression, *n* (%)^a^			
Low expression	0	5 (22.7)	5 (13.5)
Medium expression	8 (53.3)	5 (22.7)	13 (35.1)
High expression	7 (46.7)	12 (54.5)	19 (51.4)
Surgery	9 (60.0)	18 (81.8)	27 (73.0)
Median no. of previous lines, *n* (%)	2	2	2
1	3 (20.0)	3 (13.6)	6 (16.2)
2	9 (60.0)	10 (45.5)	19 (51.4)
≥3	3 (20.0)	9 (40.9)	12 (32.4)
Previous systemic therapies, *n* (%)			
Fluorouracil/analogs and derivatives	15 (100)	22 (100)	37 (100)
Taxanes	12 (80.0)	15 (68.2)	27 (73.0)
Platinum	13 (86.7)	19 (86.4)	32 (86.5)
Anti-PD-1/PD-L1 antibody	6 (40.0)	7 (31.8)	13 (35.1)
Polykinase inhibitor ^b^	4 (26.7)	7 (31.8)	11 (29.7)

GC/GEJ patient characteristics	Dose escalation/de-escalation *n* = 12	Dose-expansion *n* = 16	Total *n* = 28
Histological (Lauren) classification, *n* (%)			
Intestinal type	6 (50)	4 (25.0)	10 (35.7)
Diffuse type	3 (25)	6 (37.5)	9 (32.1)
Mixed type	2 (16.7)	5 (31.3)	7 (25.0)
Unknown	1 (8.3)	1 (6.3)	2 (7.1)
Histological (WHO) classification, *n* (%)			
Mucinous adenocarcinoma	0	1 (6.3)	1 (3.6)
Signet-ring cell carcinoma	3 (25)	9 (56.3)	12 (42.9)
Other	9 (75)	5 (31.3)	14 (50.0)
HER2 status, *n* (%)			
Negative	11 (91.7)	16 (100)	27 (96.4)
Positive	0	0	0
Unknown	0	1 (20)	1 (3.6)
Numbers of metastatic organs, *n* (%)			
≤2	5 (41.7)	9 (56.3)	14 (50.0)
≥3	7 (58.3)	7 (43.8)	14 (50.0)
Peritoneal metastases, *n* (%)	9 (95.0)	10 (62.5)	19 (67.9)
Median no. of previous lines, *n* (%)			
1	2 (16.7)	3 (18.8)	5 (17.9)
2	7 (58.3)	8 (50.0)	15 (53.6)
≥3	3 (25.0)	5 (31.3)	8 (28.6)
Previous systemic therapies, *n* (%)			
Fluorouracil/analogs and derivatives	12 (100)	16 (100)	28 (100)
Taxanes	10 (83.3)	11 (68.8)	21 (75.0)
Platinum	12 (100)	15 (93.8)	27 (96.4)
Anti-PD-1/PD-L1 antibody	6 (50.0)	6 (37.5)	12 (42.9)
Polykinase inhibitor ^b^	4 (33.3)	6 (37.5)	10 (35.7)

WHO, World Health Organization.

^a^CLDN18.2 expression level by immunohistochemical staining intensity was graded as 1+, 2+, 3+ or 0 and multiplied by the percentage of tumor cells that were positive. Low expression was defined as any intensity with a percentage of <40% or intensity 1+ with any percentage; medium expression was defined as intensity 2+ or 3+ with a percentage of 40% (inclusive) to 69%; and high expression was defined as intensity 2+ or 3+ with a percentage of ≥70%.

^b^Polykinase inhibitor: multi-target tyrosine kinase inhibitor, including apatinib, anlotinib, etc.

A total of 28 (75.7%) patients received at least one cycle of the bridging therapy regimen (Table 1), including FOLFIRI (39.3% (11/28)), nab-paclitaxel (32.1% (9/28)) or irinotecan (25.0% (7/28)) during the CT041 manufacturing period at the investigators’ discretion based on patients’ conditions (Extended Data Table 1). The median duration from apheresis to infusion was 27 days (range, 22–187 days) (Supplementary Table 2). Two patients received more than one cycle of bridging therapy because constraints related to the Coronavirus Disease 2019 pandemic delayed their prescheduled on-site infusion visits. One patient received two cycles of bridging therapy due to prolonged CT041 manufacturing. All patients underwent radiographic imaging at least 14 days after bridging therapy and before preconditioning treatment. Only three patients showed a limited decrease in tumor size (5.5−9.7%) compared to the screening visit imaging.

Thirty-five patients received fludarabine/cyclophosphamide plus nab-paclitaxel (FNC) preconditioning. Two patients with PC were preconditioned with gemcitabine instead of nab-paclitaxel (Extended Data Table 1) owing to either disease progression or persistent peripheral neurotoxicity after nab-paclitaxel as the last systemic treatment. Additional cycles of preconditioning and CT041 were administered to 18 of 37 patients (48.6%), including three patients with GC who received three cycles of preconditioning/CT041 and 15 patients (nine with GC, four with PC and two with other cancers) who received two cycles (Extended Data Table 1). The median interval from the first infusion to the second infusion, and the median interval from the second infusion to the third infusion, were 72 days (35–211) and 101 days (81–140), respectively (Supplementary Table 2).

### Safety and tolerability of CT041

The primary objective was to evaluate CT041 safety and tolerability through 28 days after the first infusion. No predefined dose-limiting toxicities (DLTs) within 28 days after the first infusion were observed in the dose-escalation/de-escalation phase. However, one patient (Pt02) at the 5.0 × 10^8^ dose level suffered a grade 4 GI hemorrhage due to rapid tumor regression after the second infusion (Table 2). As a result, the dose was decreased to 2.5 × 10^8^ and 3.75 × 10^8^ CAR T cells after discussion with the DSMC (Extended Data Fig. 1).

**Table 2:** Any grade AEs occurring in ≥25% of patients and all grade 3 or 4 AEs

Preferred term^a^, *n* (%)	Grade 3 or 4	Any
Any AE^b^	37 (100)	37 (100)
Hematology	37 (100)	37(100)
Leukopenia	31 (83.8)	37 (100)
Lymphopenia	37 (100)	37 (100)
Anemia	15 (40.5)	36 (97.3)
Neutropenia	25 (67.6)	34 (91.9)
Thrombocytopenia	6 (16.2)	22 (59.5)
GI disorders	4 (10.8)	31 (83.8)
Nausea	0	18 (48.6)
Vomiting	0	13 (35.1)
Diarrhea	0	10 (27.0)
Abdominal pain	1 (2.7)	9 (24.3)
GI hemorrhage	1 (2.7)	2 (5.4)
Gastritis erosive	1 (2.7)	1 (2.7)
Obstructive pancreatitis	1 (2.7)	1 (2.7)
Immune system disorders	1 (2.7)	36 (97.3)
CRS	0	35 (94.6)
Anaphylactic shock	1 (2.7)	1 (2.7)
Infections and infestations	1 (2.7)	5 (13.5)
Bacteremia	1 (2.7)	1 (2.7)
Other		
Protein total decreased	0	32 (86.5)
Blood albumin decreased	0	28 (75.7)
Occult blood positive	0	29 (78.4)
Alanine aminotransferase increased	3 (8.1)	21 (56.8)
Aspartate aminotransferase increased	3 (8.1)	21 (56.8)
Conjugated bilirubin increased	8 (21.6)	22 (59.5)
Prothrombin time prolonged	0	19 (51.4)
Activated partial thromboplastin time prolonged	0	19 (51.4)
Blood bilirubin increased	4 (10.8)	13 (35.1)
Hypofibrinogenemia	2 (5.4)	14 (37.8)
Hypokalemia	4 (10.8)	18 (48.6)
Hyponatremia	2 (5.4)	19 (51.4)
Lipase increased	3 (8.1)	12 (32.4)
Pyrexia	3 (8.1)	36 (97.3)
Decreased appetite	0	11 (29.7)
Rash	2 (5.4)	12 (32.4)
Edema peripheral	0	10 (27.0)
Amylase increased	1 (2.7)	3 (8.1)
Hypertension	1 (2.7)	1 (2.7)
Cardiac disorders^c^	0	12 (32.4)
Respiratory, thoracic and mediastinal disorders^c^	0	12 (32.4)

^a^Medical Dictionary for Regulatory Activities version 23.1, graded according to CTCAE version 5.0.

^b^AEs occurred in ≥25% of patients or occurred with at least one grade 3 or grade 4 AE in the patients who received CT041.

^c^Important system organ classification without specific AEs occurred in ≥25% of patients or any grade 3 or grade 4 AE.

As shown in Table 2 and Supplementary Table 3, the most frequently reported adverse events (AEs) of grade 3 or higher were preconditioning-related hematologic toxicities in 37 patients (100%), leukopenia in 31 of 37 (83.8%), neutropenia in 25 of 37 (67.6%), anemia in 15 of 37 (40.5%) and thrombocytopenia in six of 37 (16.2%), which occurred within 28 days after the first infusion and generally recovered within a median of 4–9 days (Supplementary Table 4).

**Extended Data Fig. 3:**
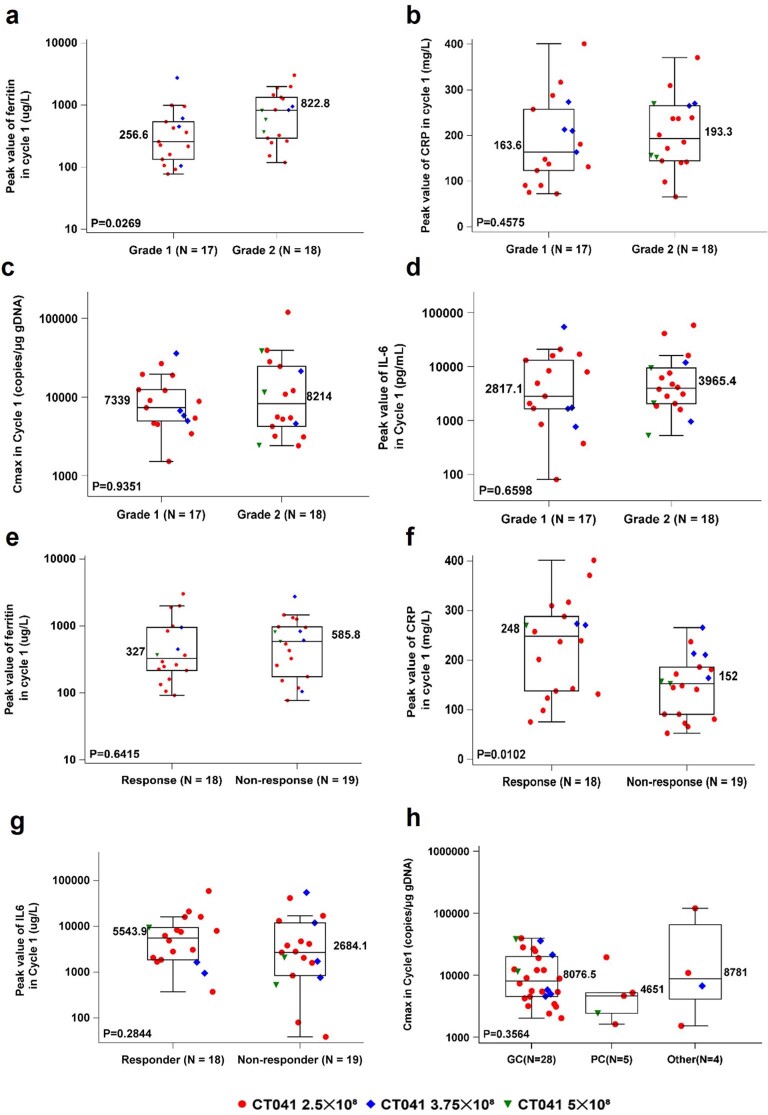
Serum biomarker peak values by tumor response and grade of cytokine release syndrome (CRS) in all patients. Relationship between **a**, post-infusion ferritin peak and CRS grade; **b**, post-infusion C-reactive protein (CRP) peak and CRS grade; **c**, maximum concentration (C_max_) of CAR-CLDN18.2 in peripheral blood and CRS grade; **d**, post-infusion IL-6 peak and CRS grade; **e**, post-infusion ferritin peak and treatment response; **f**, post-infusion CRP peak and treatment response; **g**, post-infusion IL-6 peak and treatment response. The p-values were calculated using the two-sided Wilcoxon rank sum test: **h**, The relationship between C_max_ and different cancer types. The horizontal lines and the boxes show the medians and interquartile ranges (IQR), respectively. Whiskers show the minimum observation above the lower fence (1.5 IQR below the 25^th^ percentile) and the maximum observation below upper fence (1.5 IQR above the 75^th^ percentile). The p-values were calculated using the Kruskal-Wallis (KW) test for N = 37 patients who were treated with CT041 and completed at least 12 weeks follow-up as of the data cutoff date, 08 April 2021. GC, gastric cancer; PC, pancreatic cancer.

No grade 3 or higher CRS was observed. Per the American Society for Transplantation and Cellular Therapy (ASTCT) criteria^16^, 35 of 37 (94.6%) patients had grade 1 or 2 CRS. In the first cycle of treatment, the median onset of CRS was 2 days (1–3 days) after infusion and generally lasted for a median of 6 days (3–42 days) (Supplementary Table 5). At the 2.5 × 10^8^ dose level, 26 of 28 (92.9%) patients had CRS, and 13 (46.4%) of them reported grade 2 CRS. All patients at the 3.75 × 10^8^ and 5 × 10^8^ dose levels reported CRS. Two of six (33.3%) patients at the 3.75 × 10^8^ dose level and three of three (100%) patients at the 5 × 10^8^ dose level reported grade 2 CRS. No clear dose–response relationship was observed for CRS incidence or for severity, nor was there a clear relationship between cancer type and CRS incidence or severity; however, potential associations could not be excluded owing to the limited sample size (Supplementary Fig. 2). In contrast, patients who experienced grade 2 CRS (18/35) had a higher median peak value of ferritin than those with grade 1 CRS (*P* = 0.0269; Extended Data Fig. 3a), suggesting that the peak value of ferritin could be a clinical indicator for CRS grade.

Twenty-seven patients were treated with tocilizumab and four with glucocorticoids for CRS in the first cycle (Supplementary Table 5). Per ASTCT criteria^16^, no immune effector cell-associated neurotoxicity syndrome (ICANS) was observed. No treatment-related death was reported. Four patients (4/37, 10.8%) reported reversible grade 3/4 GI AEs, including the grade 4 GI hemorrhage mentioned above and three grade 3 GI AEs of abdominal pain, obstructive pancreatitis and gastric mucosal injury (Table 2). Grade 1/2 gastric mucosal injuries were also identified in five patients who recovered after treatment with prokinetic agents and proton pump inhibitors, with a median recovery time of 19 days (10–80) (Supplementary Fig. 3 and Supplementary Table 6).

One patient developed anaphylactic shock immediately after the second infusion and recovered in 30 minutes after treatment with adrenaline and glucocorticoids. This patient had a history of multiple allergies, including allergies to previous anti-PD-1 antibody and platinum treatment. Notably, this patient’s anti-CLDN18.2 CAR antibody (anti-drug antibody (ADA)) levels remained negative at all time points, and no specific cause of the anaphylactic shock could be determined.

### Efficacy of CT041

**Fig. 1:**
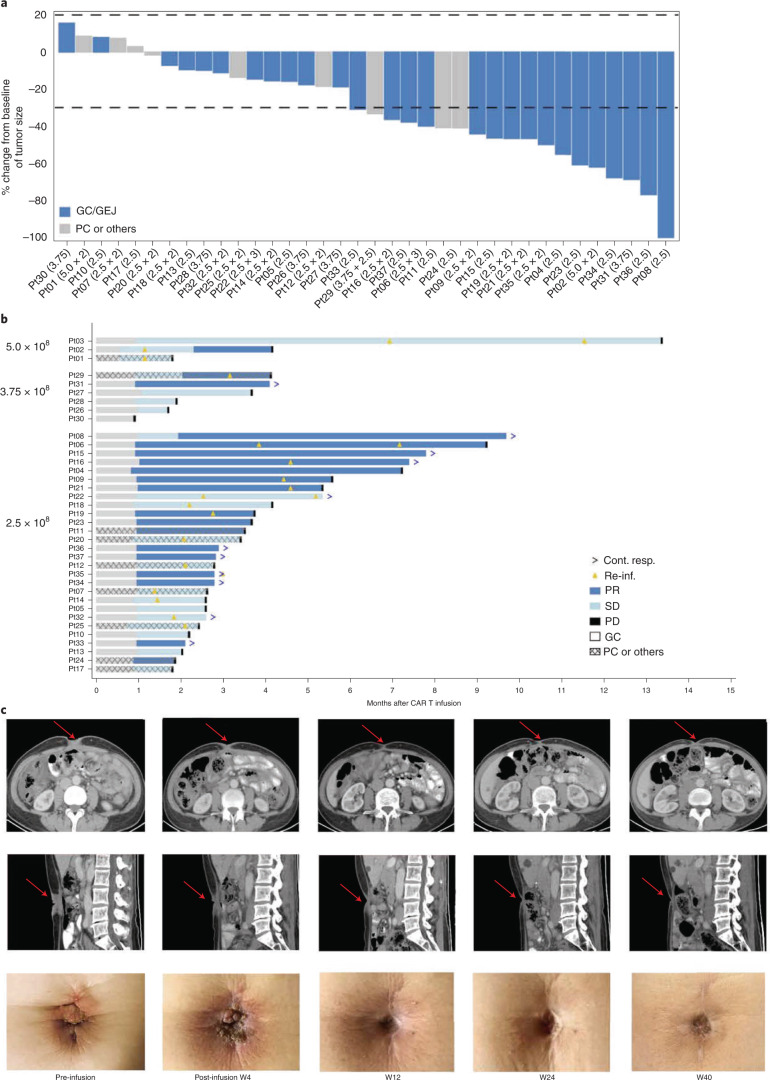
Objective responses, durations of response and representative tumor complete response images. **a**, Change in tumor size after CT041 in the 36 patients with target lesions. **b**, Best responses of each patient assessed according to RECIST 1.1 by the investigator, grouped by dose. **c**, Tumor lesion of the umbilicus in Pt08 disappeared after CT041 infusion as visualized by CT scans and photography. W, week.

**Extended Data Fig. 4:**
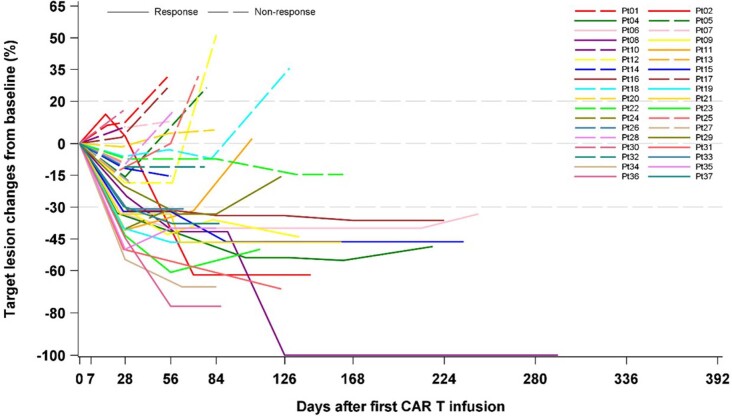
Spider plot of clinical responses. Changes in tumor size over time after CT041 infusion in the 36 patients with measurable target lesions. Solid lines indicate patients who achieved a best response of partial response or better; dashed lines indicate patients who achieved a best response of stable disease or progressive disease.

Secondary endpoints included preliminary CT041 efficacy, pharmacokinetics and immunogenicity. For preliminary efficacy, 36 of 37 patients had measurable target lesions; among them, 30 (83.3%) patients showed tumor regression (Fig. 1 and Extended Data Fig. 4). The ORR and DCR were 48.6% (95% confidence interval (CI), 31.9, 65.6) and 73.0% (95% CI, 55.9, 86.2) for all patients. The median progression-free survival (mPFS) was 3.7 months (95% CI, 2.6, 5.4) for all patients, and the overall survival (OS) rate at 6 months was 80.1% (95% CI, 62.5, 90.0; Table 3 and Supplementary Table 7).

**Table 3:** Efficacy evaluation according to tumor type*

Variable	Gastric *n* = 28	Other *n* = 9	All *n* = 37
Best overall response			
CR, *n* (%)	0	0	0
PR, *n* (%)	16 (57.1)	2 (22.2)	18 (48.6)
SD, *n* (%)	5 (17.9)	4 (44.4)	9 (24.3)
PD, *n* (%)	7 (25.0)	3 (33.3)	10 (27.0)
ORR, *n* (%) [95% CI]	16 (57.1) [37.2, 75.5]	2 (22.2) [2.8, 60.0]	18 (48.6) [31.9, 65.6]
DCR, *n* (%) [95% CI]	21 (75.0) [55.1, 89.3]	6 (66.7) [29.9, 92.5]	27 (73.0) [55.9, 86.2]
mPFS (months) [﻿95% CI]	4.2 [3.7, 9.2]	2.6 [1.8, 3.5]	3.7 [2.6, 5.4]
6-month OS rate (%) [﻿95% CI]	81.2 [60.3, 91.8]	77.8 [36.5, 93.9]	80.1 [62.5, 90.0]
6-month DOR rate (%) [﻿95% CI]	53.3 [20.7, 77.8]	NA	44.8 [17.3, 69.3]

CR, complete response; NA, not applicable.

*Tumor response was confirmed based on investigator assessment according to RECIST version 1.1.

**Fig. 2:**
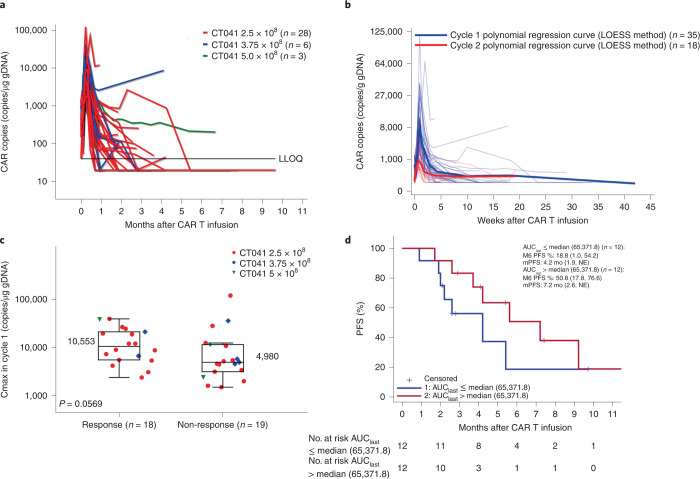
Correlation of CAR T cell expansion with dose, infusion cycle, response and type of tumor. **a**, CAR copies as measured by vector transgene copies per microgram of genomic DNA in peripheral blood, according to dose group. LLOQ, lower limit of quantitation. **b**, CAR copy numbers after first and second infusions. Bold lines: polynomial regression lines using the LOESS method. **c**, The correlation between peak vector transgene copies per microgram of gDNA after the first infusion and the occurrence of tumor response. Patients with response, *n* = 18; patients with non-response, *n* = 19. Horizontal lines and boxes show the medians and interquartile ranges (IQRs), respectively. Whiskers show the minimum observation above the lower fence (1.5 IQR below the 25th percentile) and the maximum observation below the upper fence (1.5 IQR above the 75th percentile). The *P* value was based on a two-sided Wilcoxon rank-sum test. **d**, PFS curves were estimated by the Kaplan–Meier method. *n* = 37 patients infused with CT041 who completed 12 weeks of follow-up visits for all panels. NE, not estimable.

For patients with GC, the ORR and DCR reached 57.1% (95% CI, 37.2, 75.5) and 75% (95% CI, 55.1, 89.3), respectively (Table 3 and Supplementary Table 7). The mPFS was 4.2 months (95% CI, 3.7, 9.2), and the OS rate at 6 months was 81.2% (95% CI, 60.3, 91.8). Among the 16 patients with GC who responded to CT041, 14 patients achieved partial response (PR) at week 4 after the first CT041 infusion; one patient responded at week 8; and one patient responded after the second infusion (Fig. 1b). Responders were observed to have higher peak CAR copies as well as C-reactive protein after the first infusion than those of non-responders (Fig. 2 and Extended Data Fig. 3f). As shown in Supplementary Table 7, 18 patients with GC who had failed at least two prior lines of therapy and were treated with 2.5 × 10^8^ CAR T cells achieved an ORR of 61.1%, a DCR of 83.3%, an mPFS of 5.6 months (95% CI, 2.6, 9.2) and a DOR rate at 6 months of 57.1%.

Notably, Pt08 (GC, 57 years old), who had received three prior lines of therapy, including anti-PD-1 antibody, responded to CT041 treatment as seen by the shrinkage of an umbilicus tumor lesion. The lesion achieved remission after infusion, evaluated via visual inspection and computed tomography (CT) scans from week 12 to week 40, and the response was still ongoing as of the cutoff date (Fig. 1c).

**Extended Data Fig. 5:**
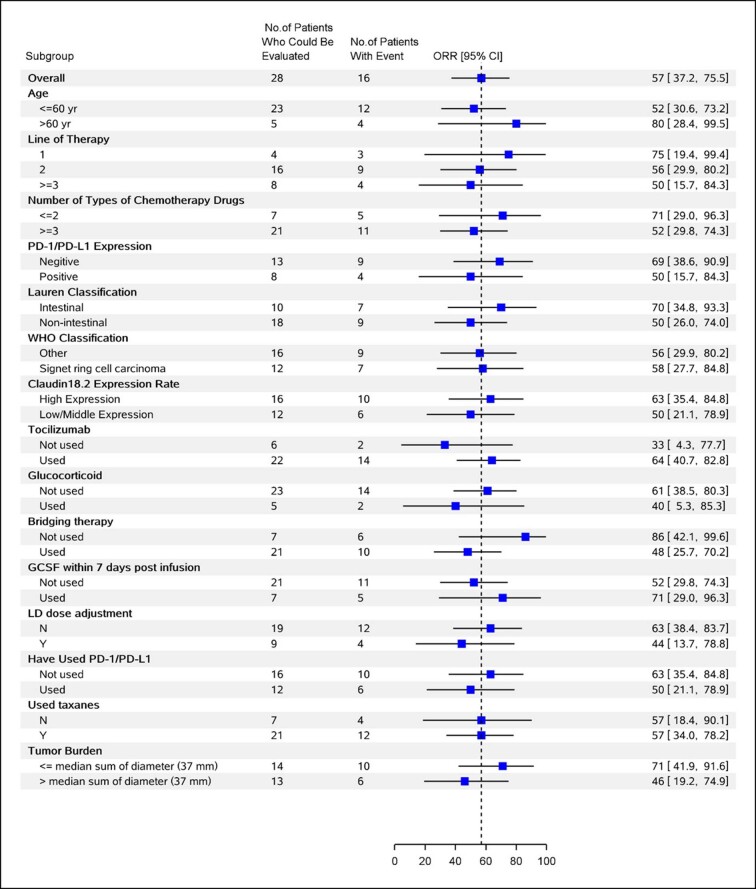
Subgroup analysis of objective response in GC patients. The objective response analysis according to key baseline and clinical covariates. The Clopper-Pearson method was used to calculate the 95% confidence interval (not adjusted for multiplicity). Squares and bars represent the ORRs and 95% CIs for each subgroup, respectively. The vertical dashed line indicates the point estimation of ORR (57%) in 28 patients with GC.

Subgroup analyses in patients with GC indicated that CT041 had an ORR of at least 40% in most subgroups (Extended Data Fig. 5), especially in patients who had failed anti-PD-1/PD-L1 antibody treatment (12/28) and/or taxane treatment (21/28). The ORR reached as high as 70% in patients with Lauren intestinal type and 63% in patients with high CLDN18.2 expression level defined as intensity 2+ or 3+ with a percentage of ≥70%. Interestingly, the ORR was numerically higher in patients who did not receive bridging therapy, which was also observed in a CD19-targeted CAR T cell trial for large B cell lymphoma^17^. The basis for this observation could be that patients who required bridging therapy could have more rapidly progressive disease (PD) than those with less aggressive disease during the CAR T cell manufacturing period.

### CT041 expansion and persistence

The median C_max_ value in the first infusion cycle was 6,713 copies per microgram of genomic DNA (gDNA) (1,519–119,581); the median T_max_ value was 7 days (1–14); and the median persistence in peripheral blood was 28 days (14–203) after the first infusion (Fig. 2a).

For the second infusion, the C_max_ significantly decreased to a median value of 1,151 copies per microgram of gDNA (334–9,877), aligning with a lower toxicity profile after the second dose, such as decreased CRS incidence (72.2%, 13/18) (Fig. 2b and Supplementary Table 5).

As shown in Fig. 2c, the C_max_ of CAR copies in the blood was higher in the responders than in the non-responders (10,553 copies per microgram of gDNA versus 4,980 copies per microgram of gDNA, *P* = 0.059). The PFS positively correlated with increased AUC_last_, which represented CAR T cell expansion and persistence (Fig. 2d).

**Extended Data Fig. 6:**
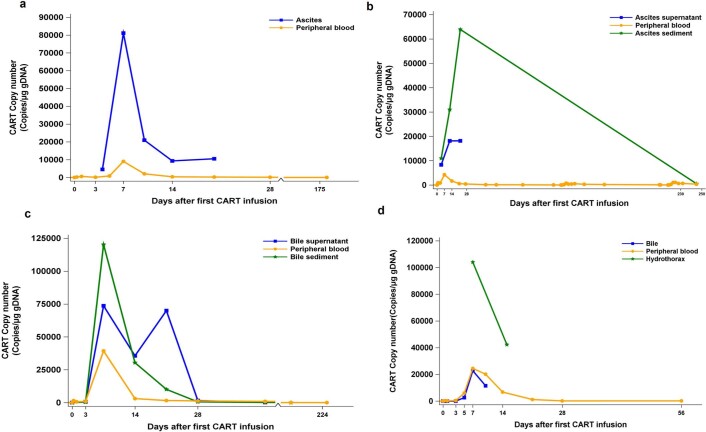
Distribution of CT041 in bodily fluids in specific patients. The distribution of CT041 for **a**, Pt 08, **b**, Pt 06, **c**, Pt 04 and **d**, Pt 23 in peripheral blood, pleural effusion, peritoneal effusion, or bile. CT041 distribution in terms of CAR-CLDN18.2 vector copies was detected by qPCR.

We explored the distribution of CAR T cells in other body fluids in four patients with GC, including bile (2/4), ascites (1/4) and pleural effusions (2/4). Compared to those in serum, the C_max_ value increased significantly, and the T_max_ value was similar (Extended Data Fig. 6).

In our study, repeated biopsies were conducted in eight patients after the first infusion, and no substantial changes in antigen expression level were observed after CT041 treatment (Supplementary Fig. 4).

### Correlation between ADAs and response

Twenty-eight of 37 patients had at least one sample with detectable ADAs after infusion, and the remaining nine patients were ADA-negative in all their visits until the data cutoff date. The ORRs were 53.6% and 33.3% in ADA-positive patients and ADA-negative patients, respectively, without statistical differences identified (*P* = 0.45) (Extended Data Table 2 and Supplementary Table 8). The sensitivities of the screening and the confirmatory ADA assay were 27.5 ng ml^−1^ and 52.6 ng ml^−1^, respectively, which were more sensitive than the recommendation of the US Food and Drug Administration (100 ng ml^−1^)^18^.

### CT041 T cell subset frequencies and clinical activity

**Fig. 3:**
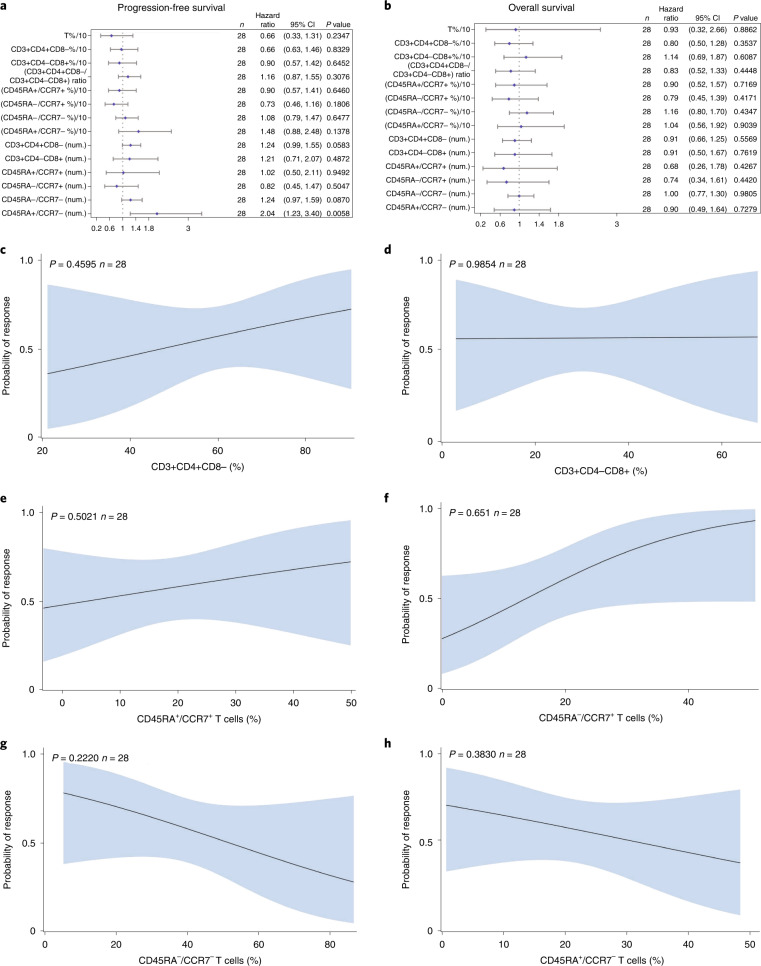
Correlations between T cell subsets in CT041 and clinical activity in patients with GC. **a**,**b**, Forest plots showing Cox regression analysis of the effect of T cell subsets on PFS and OS. **a**, Univariate analysis between PFS and infused subset cell numbers—product characteristics. **b**, Univariate analysis between OS and infused subset cell numbers—product characteristics. Dots and bars show the hazard ratios and 95% CIs for each T cell subset, respectively; *P* values were calculated using the Cox regression model, for *n* = 28 patients with GC. Logistic regression analyses were performed to determine the correlation among the frequencies of CD3^+^CD4^+^CD8^−^ (Th) cells (**c**), CD3^+^CD4^−^CD8^+^ (CTLs) (**d**), CD45RA^+^/CCR7^+^ (naive T) cells (**e**), CD45RA^−^/CCR7^+^ (central memory T) cells (**f**), CD45RA^−^/CCR7^−^(effector memory T) cells (**g**) or CD45RA^+^/CCR7^−^(terminally differentiated effector T) cells (**h**) in CT041 product to probability of response. CT041 was evaluated before infusion. Shaded bands show the 95% CIs of predicted probability, and lines present the predicted probability of response from logistic regression analyses with nominal *P* values (that is, without adjustment for multiplicity), for *n* = 28 patients with GC.

The correlation between T cell subset frequencies and clinical activity was an exploratory endpoint. We evaluated the T cell subsets, including CD3^+^CD4^+^CD8^−^ (Th) cells, CD3^+^CD4^−^CD8^+^ (cytotoxic T lymphocytes (CTLs)), CD45RA^+^/CCR7^+^ (naive T) cells, CD45RA^−^/CCR7^+^ (central memory T) cells, CD45RA^−^/CCR7^−^ (effector memory T) cells and CD45RA^+^/CCR7^−^ (terminally differentiated effector T) cells for all the patients before infusion and analyzed the correlation between T cell subset frequencies and CT041 anti-tumor activity in 28 patients with GC. Patients infused with CT041 that contained a lower frequency of CD45RA^+^/CCR7^−^ T cells were more likely to achieve longer PFS (*P* = 0.0058) (Fig. 3a). No statistically significant correlation was observed between any of the T cell subsets and OS (Fig. 3b). Frequencies of T cell subsets in CT041 products showed no statistically significant association with clinical response (Fig. 3c–h).

**Extended Data Fig. 7:**
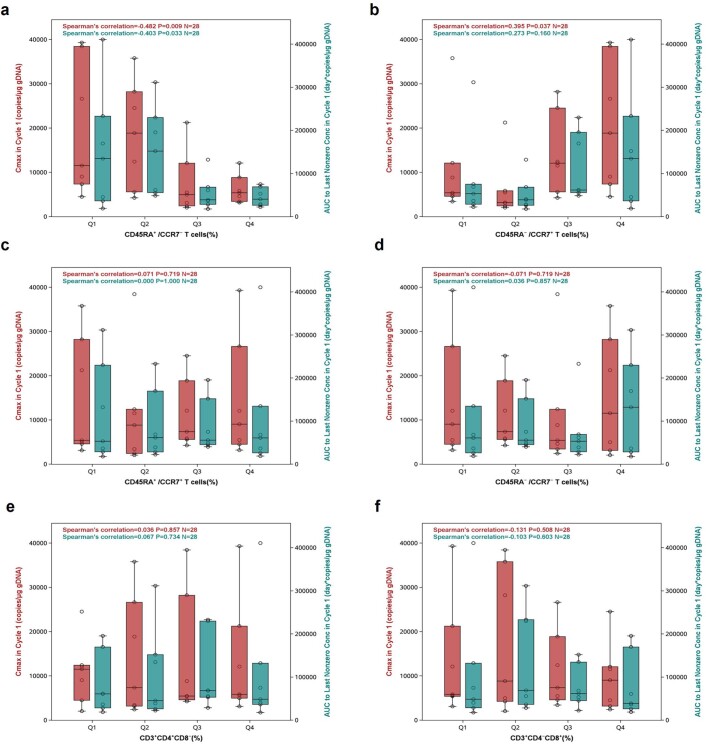
Correlation between frequencies of T-cell subsets and C_max_ or AUC_last_ of CAR copies after first infusion in GC patients. Logistic regression analysis of C_max_ or AUC_last_ of CAR copies after first infusion and the frequencies of, **a**, CD45RA + /CCR7- (terminally differentiated effector T) cells, **b**, CD45RA-/CCR7 + (central memory T) cells, **c**, CD45RA + /CCR7 + (naive T) cells, **d**,CD45RA-/CCR7- (effector memory T) cells, **e**, CD3 + CD4 + CD8^−^ (Th) cells, or **f**, CD3 + CD4^−^CD8 + (cytotoxic T lymphocytes) cells in CT041 product.

We also analyzed the correlation between frequencies of T cell subsets and C_max_ or AUC_last_ of CAR copies after the first infusion. The results revealed that CT041 products containing lower frequencies of CD45RA^+^/CCR7^−^ cells achieved higher C_max_ and AUC_last_ (*P* = 0.009 and *P* = 0.033; Extended Data Fig. 7a). The frequency of CD45RA^−^/CCR7^+^ cells was positively correlated with C_max_ (*P* = 0.037; Extended Data Fig. 7b).

## Discussion

The results of the present study revealed that CT041 was well-tolerated in patients with CLDN18.2-positive cancers of the digestive system. No DLTs (within 28 days after the first infusion), treatment-related deaths or AEs leading to study withdrawal were reported. All patients experienced expected transient hematologic toxicities mainly related to the preconditioning regimens. No difference was found in AE incidence between different dose levels. In terms of on-target off-tumor toxicity, only one grade 3 mucosal erosion was reported in one patient with PC. Although CLDN18.2 is highly expressed in short-lived differentiated gastric mucosal cells^8^, manageable mucosal injury AEs were reported in only a minority (6/37) of the patients, of which five were grade 1 or grade 2. Our preclinical study indicated that NOD/SCID mice treated with CLDN18.2 CAR T cells displayed no obvious toxicities in normal stomach tissues^12^, which might be ascribed to the limited exposure of CAR-binding epitope in normal tissues, the normal tissue microenvironment or the rapid tissue repair capacity of CLDN18.2-negative stomach normal stem cells.

In prior clinical reports, nivolumab (ATTRACTION 2 study), pembrolizumab (KEYNOTE-059 study, cohort 1), trifluridine/tipiracil (TAGS study) and apatinib (a small-molecule VEGFR inhibitor developed mainly in China), which had been administered in the third-line setting for GC, achieved ORRs of 11.2%, 13.3%, 4% and 1.7%, respectively^19–22^. The mPFSs for these treatments were limited at 1.6–2.6 months, and OSs were no more than 6 months^19–22^. Compared to the aforementioned therapies, CT041 achieved promising results in the third-line setting for GC. Moreover, the patients with GC in this study had tumors pathologically characterized as 42.9% signet-ring cell carcinoma and 57.1% Lauren diffused/mixed type, which are regarded as poor prognostic subtypes of advanced GC^23,24^. In contrast, the KEYNOTE-059 study treated only 2.7% patients with signet-ring cell carcinoma. In addition, patients with GC and peritoneal metastases have an overall survival ranging from 2 months to 9 months^25^, although advances in combination intraperitoneal and systemic chemotherapy administration may offer future improvement^26^. This may, in part, be due to limited penetration of systemic administration of anticancer drugs to peritoneal carcinomas^27^. Conversely, despite the intravenous CT041 infusion, we surprisingly observed much higher CAR T cell expansion in ascites than in peripheral blood of patients with GC with multiple peritoneal metastases. In our study, 67.9% of patients with GC were diagnosed with peritoneal metastasis on baseline CT imaging, in contrast to the markedly lower percentages of 1.5%, 26% and 24.4% in the third-line setting studies of KEYNOTE-059, TAGS and apatinib, respectively^20–22^. In short, it is encouraging for CT041 to achieve such promising efficacy in the subgroup of patients with refractory advanced GC compared to currently available treatment options. In addition, CAR T cell expansion and/or accumulation in ascites might be advantageous for eliminating diffuse peritoneal metastatic lesions, which warrants further investigation.

Previous studies indicated that the response rate of anti-PD-1/PD-L1 antibody might be affected by the tumor immune microenvironment^28–30^. Interestingly, we observed nearly 70% ORR in patients without PD-L1 expression, and six of 12 patients who had failed anti-PD-1/PD-L1 antibody treatment responded to CT041 (Extended Data Fig. 5). These data suggest that the efficacy of CAR T cell therapy might not be limited by PD-L1 expression status or impaired by previous use of anti-PD-1/PD-L1 blockade. Therefore, CT041 might potentially bring clinical benefits to patients who have no effective treatments.

In this study, 75% of the patients with GC received bridging therapy (Table 1), and only three patients had a modest (<10%) tumor reduction before preconditioning. The results suggest that the objective response after CT041 treatment should mainly be ascribed to CT041, although the potential contribution from bridging therapy and preconditioning regimen could not be fully excluded. Preconditioning chemotherapy with fludarabine and cyclophosphamide is commonly used for CD19 CAR T cell therapy; however, this regimen has low efficacy for cancers of the digestive system^13^. Thus, this regimen may not effectively improve the tumor microenvironment and help the infiltration and function of CAR T cells in solid tumors. Preclinical and clinical studies have shown that nab-paclitaxel can be transported across the endothelial cells, accumulate in the tumor stroma and disrupt cancer–stromal interactions^31–33^. Administration of low-dose nab-paclitaxel significantly decreased the accumulation and immunosuppressive activities of tumor-associated myeloid-derived suppressor cells^34^. Therefore, a non-cytotoxic dose of nab-paclitaxel may improve the infiltration and function of CAR T cells. In this study, we used a single 100-mg fixed dose of nab-paclitaxel, which was much lower than the standard pharmacological dose^35,36^. Despite the low dose of nab-paclitaxel, the subgroup analyses showed that 21 of 28 patients with GC who had failed taxane treatment, including six who had received nab-paclitaxel, achieved a high response rate. Because we observed preliminary good safety and promising efficacy results for CT041 with the FNC regimen, the FNC regimen was recommended in the protocol as the preconditioning regimen. Although it will be important to explore if/how the FNC regimen facilitates the high response of CT041, the requisite repeated biopsies before and after preconditioning would be challenging to perform owing to ethical considerations.

The expansion and persistence of CT041 was generally consistent with the response rate and PFS. Because no considerable difference in cell expansion was observed among the three dose levels, and a similar response rate was observed for the dose of 2.5 × 10^8^ cells compared to other dose levels, this dose was recommended for further development in the expansion cohort after consulting with the DSMC. The median persistence of CT041 in peripheral blood after the first infusion was 28 days, shorter than that of CAR T cells in hematologic malignancies. CAR T cell persistence in peripheral blood is potentially associated with duration of tumor response in hematologic malignancies. However, the expansion and persistence of CAR T cells within solid tumor tissues are difficult to evaluate. We did observe durable responses in a few patients after CT041 was undetectable by qPCR in the peripheral blood. Future studies are needed for a better understanding of the mechanisms underlying response durability.

According to previous reports, CD19 epitope loss is a relatively common tumor escape mechanism after treatment with CD19-redirected CAR T cell therapy^37,38^. In contrast, BCMA antigen loss is an uncommon mechanism for relapsed or refractory multiple myeloma^4^. In this study, the CLDN18.2 expression levels were not downregulated after CT041 infusion (Supplementary Fig. 4), suggesting that antigen escape may not be the common mechanism of acquired resistance against CT041, which deserves further investigation.

Interestingly, our data showed that a lower frequency of CD45RA^+^/CCR7^−^ terminally differentiated effector T cells in CT041 was more likely to achieve longer PFS and higher C_max_ and AUC_last_. Additionally, we observed a positive correlation between the frequency of CD45RA^−^/CCR7^+^ central memory T cells and C_max_. These data suggest that the T cell phenotype composition of CT041 may be critical for its efficacy. Furthermore, previously reported data indicated that naive T cells and central memory T cells could drive anti-tumor activity^39^. More data are needed to investigate the association between CAR T cell subsets and treatment efficacy.

Taken together, these interim results indicate that CT041 has the potential to become an important treatment modality for patients with advanced GC, suggesting that CAR T cell therapy could benefit not only patients with hematologic malignancies but also those with solid tumors. CT041 showed a trend of more benefit in patients with higher intratumoral CLDN18.2 expression. These initial observations remain to be verified in the complete trial. In addition to GC, further investigation is needed to fully understand the characteristics of patients with sustained responses in other GI tumor types.

## Methods

### Study design and number of patients

This open-label, phase 1 trial was conducted in three centers in China. It consists of two phases: a modified ‘3 + 3’ pattern dose-escalation/de-escalation phase and a dose-expansion phase. In the dose-escalation/de-escalation phase, four dose levels were planned: 5.0 × 10^8^ CAR T cells as an initial dose, 10.0 × 10^8^ CAR T cells, 2.5 × 10^8^ CAR T cells and 3.75 × 10^8^ CAR T cells. After the recommended dose was obtained in the dose-escalation/de-escalation phase, patients with CLDN18.2 expression positive were screened for the dose-expansion study. A total of 12–24 patients (3–6 patients in each dose level) in the dose-escalation/de-escalation phase, followed by 15–25 patients in the expansion phase, were planned by the data cutoff date (Extended Data Fig. 1).

The trial was initiated by the investigators. The protocol was approved by the local ethics committee of the Department of Gastrointestinal Oncology, Peking University Cancer Hospital (2018YJZ75). All patients provided written informed consent. This study was conducted in accordance with the International Conference on Harmonization Guidelines for Good Clinical Practice and the Declaration of Helsinki. Patients received compensation for travel and a meal per visit.

### Patient eligibility

Patients eligible for inclusion in this study had to meet all the following criteria:

1. Age ≥18 and ≤75 years, male or female.
2. Histologically confirmed solid tumor (GC/GEJ cancer, PC or other digestive system malignancies) who have failed at least one line of treatment.
3. IHC staining of tumor tissue samples must be positive for CLDN18.2 (expression intensity ≥2+ and positive tumor cell rate ≥40%).
4. Estimated life expectancy >12 weeks.
5. According to Response Evaluation Criteria in Solid Tumors (RECIST) version 1.1, there are measurable lesions or non-measurable lesions.
6. ECOG performance status score of 0 or 1 within 24 hours before apheresis and before preconditioning.
7. Adequate venous access for peripheral blood mononuclear cell (PBMC) collection.
8. Patients must meet the following criteria at screening and before preconditioning (baseline). If any laboratory test result is abnormal referring to the following criteria, it is acceptable to test one more time within 1 week. If the test result is still abnormal, the patient is screen failed:
  - Hematology (no intensive blood transfusion (≥2 times within 1 week), platelet transfusion or cell growth factor (except for recombinant erythropoietin) performed within 7 days before the test): neutrophils (NE) ≥1.5 × 10^9^ per liter, lymphocytes (LY) ≥0.5 × 10^9^per liter (except for before preconditioning), platelets (PLT) ≥75 × 10^9^per liter and hemoglobin (Hb) ≥8.0 g dl^−1^. Patient also must meet these criteria within 24 hours before apheresis.
  - Blood chemistry: creatinine clearance ≥40 ml min^−1^ (Cockcroft–Gault formula), alanine aminotransferase (ALT) ≤2.5 × ULN, aspartate aminotransferase (AST) ≤2.5 × ULN, total bilirubin (TB) ≤2 × ULN, serum lipase and amylase <1.5 ULN, alkaline phosphatase (ALP) ≤2.5 ULN; for patients with bone or hepatic metastasis, AST, ALT and ALP <5 ULN.
  - Prothrombin time ≤ULN +4 seconds.
9. Women of childbearing potential must have negative serum pregnancy test result at screening and before preconditioning and agree to use an effective and reliable contraceptive method for at least 1 year after the last study treatment. The acceptable methods include bilateral tubal ligation/bilateral salpingectomy or bilateral tubal occlusion; any approved oral, injection or implantation of hormone; or barrier contraceptive method: condoms containing spermicidal foam/gel/film/paste/suppositories or occlusive cap (diaphragm or cervical/fornix cap).
10. Male patients who have not undergone vasectomy and have sexual activity with women of childbearing potential must agree to the use of a barrier contraceptive method—for example, condoms containing spermicidal foam/gel/film/paste/suppositories or the use of contraceptive methods to their partner (see inclusion criteria, no. 9). Sperm donation is prohibited within 1 year after the last study treatment.
11. Voluntarily willing to participate in the study and sign the written informed consent.

Patients are not eligible to be included in the study if they meet any of the following criteria:

1. Pregnant or lactating women.
2. HIV, treponema pallidum or HCV serology is positive.
3. Patients with any uncontrolled active infection, including, but not limited to, active tuberculosis or HBV infection (HBsAg positive or HBV DNA positive).
4. Patients who have thyroid dysfunction according to serum thyroid hormone assay results (total thyroxine (TT4), total triiodothyronine (TT3), free triiodothyronine (FT3), free thyroxine (FT4) and serum thyroid stimulating hormone (TSH)) and the patient is not suitable to participate in the study according to the investigator’s assessment.
5. Patients with AEs induced by previous treatment that have not recovered to Common Terminology Criteria for Adverse Events (CTCAE) ≤1, except for alopecia, chromatosis and other tolerable events judged by the investigator or permitted laboratory abnormalities according to the protocol.
6. Systemically glucocorticoid medicine ≥15 mg per day within 7 days before apheresis; inhalations containing corticosteroids are not excluded.
7. Patient allergic or intolerant to preconditioning drugs, including, but not limited to, fludarabine and cyclophosphamide or tocilizumab; allergic to the components of CT041; penicillin allergy history confirmed by positive skin test; or any severe allergy history—for example, anaphylactic shock.
8. Any previous treatment of genetically engineered modified T cell therapy (including CAR-T and TCR T cell) other than this product in the past.
9. Patients with untreated brain metastases or symptoms of brain metastases.
10. Patients with central or extensive lung metastases or extensive hepatic metastases; more rapid tumor progression before preconditioning (at baseline) in comparison to the screening period judged by the investigator.
11. The largest target lesion is >4 cm.
12. Patients with unstable or active ulcer or active Gl tract bleeding at present.
13. Patients who have a history of organ transplantation or are waiting for organ transplantation.
14. Patients requiring anticoagulant therapy (for example, warfarin or heparin).
15. Patients requiring long-term anti-platelet therapy (aspirin > 300 mg per day or clopidogrel >75 mg per day).
16. Patients who have undergone major surgery or severe trauma within 4 weeks before apheresis or plan to have major surgery during this study.
17. Patients with other serious diseases that may restrict them from participating in this study, such as poorly controlled diabetes (glycosylated hemoglobin HbA1c >8% undertreatment), poorly controlled hypertension judged by the investigator (blood pressure >160 mmHg/100 mmHg), severe cardiac insufficiency (left ventricular ejection fraction <50%), myocardial infarction or unstable arrhythmia or unstable angina pectoris, pulmonary embolism, chronic obstructive pulmonary disease, interstitial lung disease or clinically significant lung function test abnormalities in the past 6 months.
18. Patients who are unable or unwilling to comply with the clinical protocol, by the investigator’s judgment.
19. Before apheresis and preconditioning blood oxygen saturation ≤95% (finger oxygen detection) is acceptable.
20. Before apheresis and preconditioning, patients who have the following conditions, including, but not limited to: new-onset arrhythmia that cannot be controlled by medications; hypotension that requires the use of vasopressors; or bacterial, fungal or viral infections that require intravenous antibiotic, antiviral or antifungal treatment, and the investigator judged that they are not suitable to continue the experiment. Patients who use antibiotics to prevent infection can continue the study upon the judgment of the investigator.
21. Before apheresis and preconditioning, any signs of central nervous system disease or clinically significant abnormal neurological examination results.
22. Patient with other malignant tumors within the past 3 years or at the present, except for cervical cancer in situ and basal cell carcinoma of the skin.

### CT041 manufacturing, preconditioning and bridging therapy

The collected PBMCs were further isolated and purified by Ficoll density gradient centrifugation at the CARsgen Therapeutics manufacturing site in Shanghai, China. Then, the PBMCs were seeded in culture medium supplemented with human IL-2. Dynabeads CD3/CD28 were used for T cell activation. After activation, the T cells were transduced with a lentiviral vector encoding the CAR-CLDN18.2 (ref. ^12^). The CAR T cells were harvested and formulated after the cell quantity reached the dose requirement. In-process and release tests were performed for each lot of CT041 cells. After release, the patients received the first infusion of CT041 (day 0, week 0) within 7 days after the completion of preconditioning therapy as the first cycle of treatment. Non-steroidal anti-inflammatory drugs and anti-histamines were given within half an hour before cell infusion. Re-infusion was allowed if patients met criteria, including, but not limited to: the patient achieved tumor response of stable disease (SD) or above; any severe toxicities related to study treatment recovered to grade 1 or lower or returned to baseline; and CAR-CLDN18.2 copies were undetectable in the peripheral blood before re-infusion. Patients received a second course of preconditioning chemotherapy before re-infusion (Extended Data Fig. 1).

Preconditioning therapy was administered as follows: fludarabine 25 mg m^−^^2^ on days –4 and –3; cyclophosphamide 250 mg m^−^^2^ on days –4, –3 and –2; and nab-paclitaxel 100 mg or gemcitabine 1,000 mg on day –3.

For patients whose tumor burden was heavy, or who had the potential to rapidly progress according to the investigators’ discretion during the period of CT041 manufacturing, bridging chemotherapy was allowed. The recommended treatment regimen was FOLFIRI (5-FU 2,400 mg m^−^^2^, irinotecan 180 mg m^−^^2^). Investigators could also individualize the regimen according to the patient’s previous anti-tumor therapy and the patient’s clinical condition.

### CLDN18.2 immunohistochemistry assay

Immunohistochemical studies were performed on 3~5-mm-thick sections of formalin-fixed paraffin-embedded tumor tissue. Automated immunostaining was performed using the Leica BOND-III (Leica Biosystems) in the bio-sample analysis laboratory of CARsgen Therapeutics with the validated staining method. Slides were stained with antibodies against CLDN18.2 (clone 14F8, prediluted mouse monoclonal antibody, CARsgen made in-house) with Bond Polymer Refine Detection (Leica Biosystems) and counterstained with hematoxylin according to the manufacturer’s instructions^40,41^. CLDN18.2 protein expression was determined using both the staining intensity and the percentage of stained tumor cells (the number of CLDN18.2 staining cells / the total number of viable tumor cells × 100%). Low expression was defined as any intensity with percentage <40% or intensity 1+ with any percentage; medium expression was defined as intensity 2+ and 3+ with percentage from 40% (inclusive) to 69%; and high expression was defined as intensity 2+ and 3+ with percentage ≥70%.

### Flow cytometry of CAR T cell products

Standard staining and flow cytometry techniques were used to perform immunophenotyping of surface markers on all CT041 cell products at the CARsgen Therapeutics manufacturing site (Supplementary Fig. 5). Th and CTL cells were stained with CD3-PerCP, CD4-FITC- and CD8-PE. The frequencies of naive T cells, central memory T cells, effector memory T cells and terminally differentiated effector T cells were defined by CD197-BV421 and CD45RA-FITC^39^. All antibodies for analysis were purchased from BD Biosciences. Samples (3.0 × 10^5^ cells) were stained in the dark for 15 minutes at 4 °C. Then, the cells were washed twice in PBS containing 1.0% FBS. Samples were analyzed on a FACSCanto II (BD Biosciences) flow cytometer, and the data were analyzed using FlowJo software version 10.5 (Tree Star).

### qPCR analysis of CAR CLDN18.2 cell expansion and persistence

Persistence of CAR-CLDN18.2 cells in peripheral blood was determined at the central laboratory of CARsgen Therapeutics by quantification of the woodchuck hepatitis post-transcriptional regulatory element region of the lentiviral transgene by qPCR (Supplementary Table 9)^42^. Patient samples of 10 ml of peripheral blood were collected in K2EDTA tubes at baseline and after infusion. gDNA was extracted using the QIAamp DNA Midi Kit (Qiagen). The standard curve for the transcript copy number was established by the amplification of a ten-fold serially diluted linearized plasmid PSD001 between 2 × 10^6^ and 200  copies. The number of transgene copies per microgram of gDNA was determined on a 7500 Fast (Thermo Fisher Scientific) triplicated for each sample. The limit of detection of this assay was 40 copies per microgram of gDNA.

### ADA assay

ADAs to CAR-CLDN18.2 cells were detected at the central laboratory of CARsgen Therapeutics using the human K2EDTA plasma sample with a validated electrochemiluminescence (ECL) assay on MESO QuickPlex SQ 120 (Meso Scale Discovery (MSD)). This assay detects anti-CT041 antibody by measuring the light emission by SULFO-TAG label. All the samples were duplicated with this three-tier assay, which includes screening, confirmatory and titer assays^43^.

A 1-ml aliquot of the patient plasma sample was transferred into a K2EDTA tube at baseline and days 28, 56, 84, 126, 168, 280 and 392 after infusion. Standard MSD 96-well plates were coated with the solution of 100 μl per well of 0.5 ug ml^−1^ of capture drug (hu8E5–2I scFv) in PBS at room temperature for 120 minutes. The samples and positive controls (goat anti-hu8E5–2I scFv polyclone antibody) were diluted by adding following components into a Nunc 96-well conical-bottom polypropylene plate (Thermo Fisher Scientific) and mixed: 50 μl per well of the samples and 200 μl per well of Super Block Blocking Buffer (Thermo Fisher Scientific). After blocking and washing the standard MSD 96-well plates, 100 μl of the samples and controls were transferred from the Nunc plate to the standard MSD 96-well plate and incubated at room temperature for 60 minutes. After plate washing, 100 μl per well of detection drug (SULFO-TAG-hu8E5–2I scFv) was added and incubated at room temperature for 60 minutes. After plate washing, 150 μl per well of 2× MSD Read Buffer was added, and the ECL signal responses were quantified in a Meso Scale QuickPlex 120 MSD reader. The sensitivity of the screening and confirmatory assays were 27.5 ng ml^−1^ and 52.6 ng ml^−1^, respectively.

### Outcomes

The primary endpoints were safety and tolerability during the 4 weeks after the first CT041 infusion. Secondary endpoints were pharmacokinetics of CT041, 12-month safety and tolerability and anti-tumor efficacy. Pharmacokinetics was presented with the data of CAR-CLDN18.2 DNA in peripheral blood, which was detected by qPCR at each visit since infusion. CRS and ICANS events were graded per the ASTCT criteria^18^. The number of participants with treatment-related AEs (including the AEs related to preconditioning or CT041) was assessed by CTCAE version 5.0. The anti-tumor efficacy was measured according to RECIST version 1.1. The long-term survival follow-up was a maximum of 15 years after the first infusion. Exploratory endpoints were the effect of disease characteristics, such as expression of CLDN18.2, metastases site and Lauren classification, on the efficacy of CT041 and expansion of CT041 cells after infusion.

### DSMC

A DSMC consisting of two clinical experts and one statistical expert was organized for this study. DSMC meetings were held regularly to perform unblinded data review to identify potential risks for patients. Besides the regular meetings, a DSMC meeting must be held before the study proceeds with administering the next dose level.

### Statistical analysis

The sample size was based on clinical considerations and a modified ‘3 + 3’ dose-escalation/de-escalation design. Descriptive statistics include the number of cases, mean, median, standard deviation, minimum and maximum values for continuous variables and frequency distributions for categorical variables. All AEs were categorized according to the ICH MedDRA codes version 23.1, graded according to CTCAE version 5.0 and analyzed via frequency distributions, tables or other descriptive indicators. The number and percentage of patients experiencing a treatment-emergent AE were calculated based on system organ classification, preferred term and different groups. Tables containing CAR-CLDN18.2 copy numbers at specified time points were generated. Exact methods contain the Clopper–Pearson method with 95% CIs for ORR and DCR and the Kaplan–Meier method for mPFS, DOR rate and OS rate. Logistic regression was used to explore the relationship between the covariate and tumor response. The predictive curve based on the univariate logistic regression calculation visualized the trend. The Cox regression model was used to analyze the potential correlation of covariate and time-to-event endpoints and to estimate the hazard ratios and 95% CIs.

### Reporting Summary

Further information on research design is available in the Nature Research Reporting Summary linked to this article.

## Online content

Any methods, additional references, Nature Research reporting summaries, source data, extended data, supplementary information, acknowledgements, peer review information; details of author contributions and competing interests; and statements of data and code availability are available at 10.1038/s41591-022-01800-8.

## Supplementary information

Supplementary Information 16 pages; Supplementary figures 1–5, Supplementary tables 1–9

Reporting Summary

## Figures and Tables

**Extended Data Table. 1:**
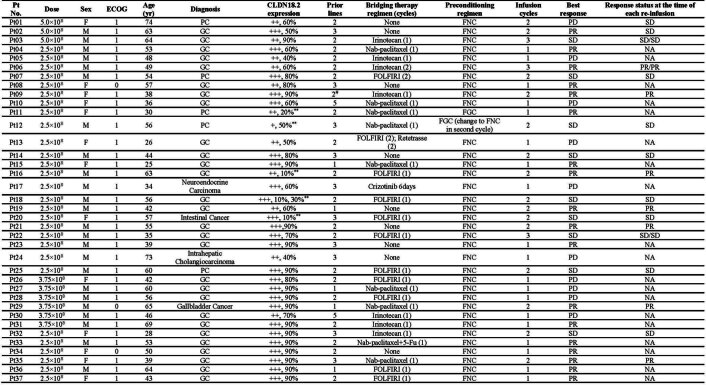
Patient level baseline, treatment and response characteristics (modified intention-to-treat analysis set)

Data are from the first 37 patients who were treated with CT041 and completed at least 12 weeks follow-up as of the data cutoff date, 08 April 2021. FGC: fludarabine + gemcitabine+ cyclophosphamide; FNC: fludarabine + nab-paclitaxel + cyclophosphamide; GC: gastric cancer including gastroesophageal junction carcinoma; NA: not applicable; PC: pancreatic cancer; PD: progressive disease; PR: partial response; SD: stable disease. #Three drug regimen including 5-Fu, platinum, and paclitaxel was considered as prior 2 lines. ^**^Patients with CLDN18.2 intensity ≤ 2+ or percentage ≥ 40% were enrolled according to protocol version 6.1, in which the inclusion criteria was CLDN18.2 expression intensity ≥ 1+ and percentage ≥10%.

**Extended Data Table 2:**
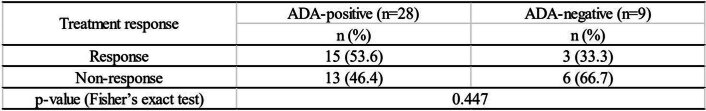
Association between patient ADA status and tumor response

Response defined as patients who achieved a best response of partial response or better; non-response defined as patients who achieved a best response of stable disease or progressive disease.

## Data Availability

All data used in the interim analyses supporting the findings of the present study are available within the manuscript and its supplementary information files. All requests for further data sharing will be reviewed by the leading clinical center, by the Department of Gastrointestinal Oncology, Peking University Cancer Hospital and Institute, and by the study collaborator, CARsgen Therapeutics Co., Ltd., to verify whether the request is subject to any intellectual property or confidentiality obligations. Figures 1a–cand 2a–c, Extended Data Table 1 and Extended Data Figs. 3, 4, 6 and 7 present individual participant-level data with privacy information de-identified to support understanding of the study results. Further requests for access to the individual participant-level data from this study can be submitted via email to the corresponding author with detailed proposals. Each participant’s rights and privacy are key subjects to take into consideration when sharing information. A signed data access agreement with the collaborator is required before accessing shared data.
