# Supplementary material for: Claudin18.2-specific CAR T cells in gastrointestinal cancers: phase 1 trial interim results
Source: Nat Med. 2022 May 9;28(6):1189–98. doi: 10.1038/s41591-022-01800-8 (PMC9205778; doi:10.1038/s41591-022-01800-8)
Supplement: Supplementary file 1 — 16 pages; Supplementary figures 1–5, Supplementary tables 1–9 [file 41591_2022_1800_MOESM1_ESM.pdf]

nature portfolio

https://doi.org/10.1038/s41591-022-01800-8

Supplementary information

Claudin18.2-specific CAR T cells in gastrointestinal cancers: phase 1 trial interim results

In the format provided by the authors and unedited

Supplementary Information

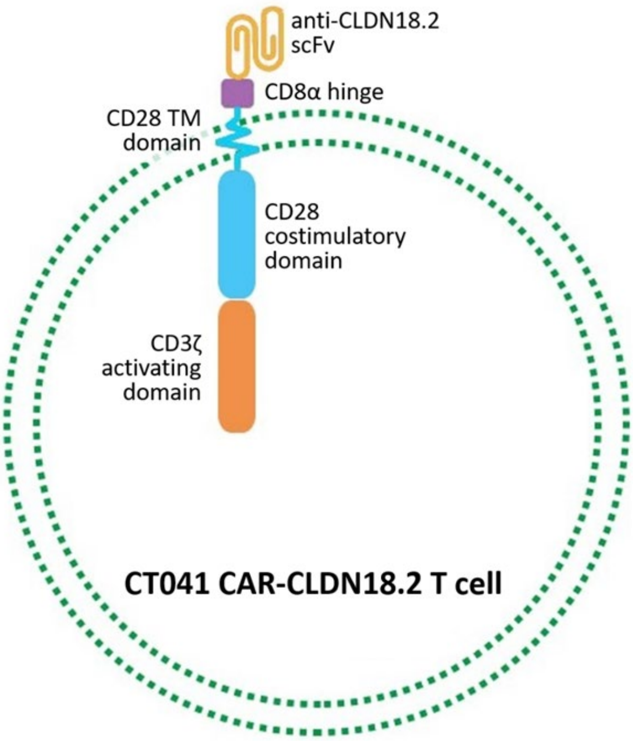

Fig. S1 | CT041 Cell Structure Diagram

CT041 is an autologous CLDN18.2-redirected CAR T-cell therapy. The chimeric antigen receptor of CT041 comprises a humanized anti-CLDN18.2 single-chain variable fragment, a CD8α hinge region, a CD28 costimulatory domain and CD3ζ signaling domain.

a

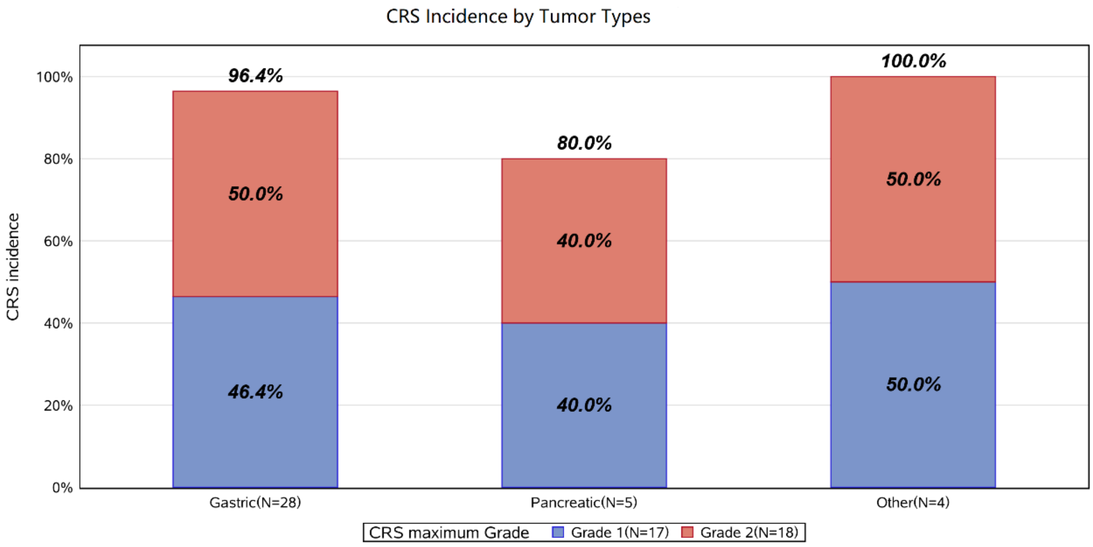

b

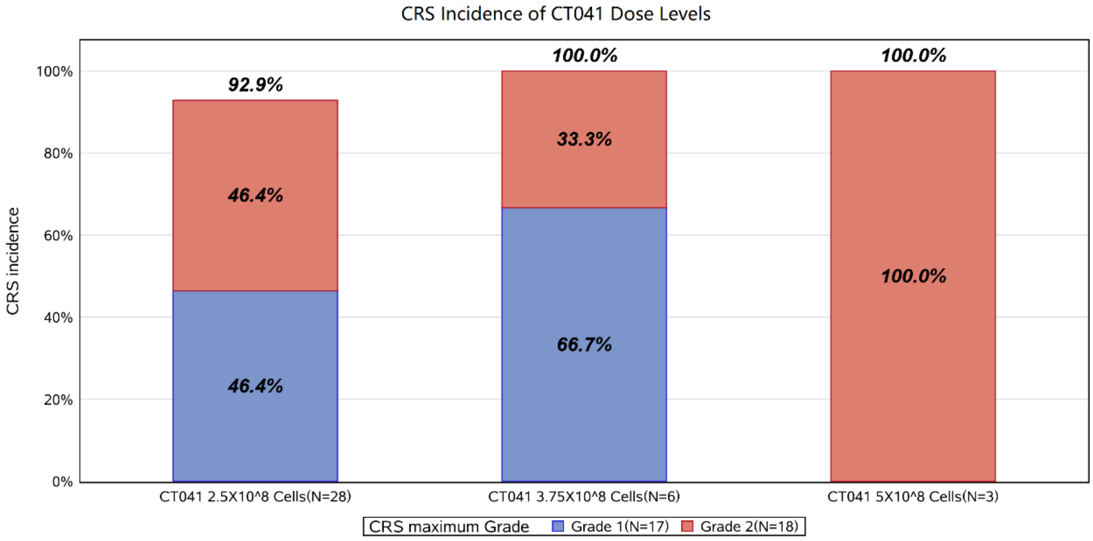

Fig. S2 | Relationship Between Cytokine Release Syndrome (CRS) Incidence, Tumor Types, and CT041 Dose Levels

a, CRS incidence by tumor types. **b**, CRS incidence by CT041 dose level.

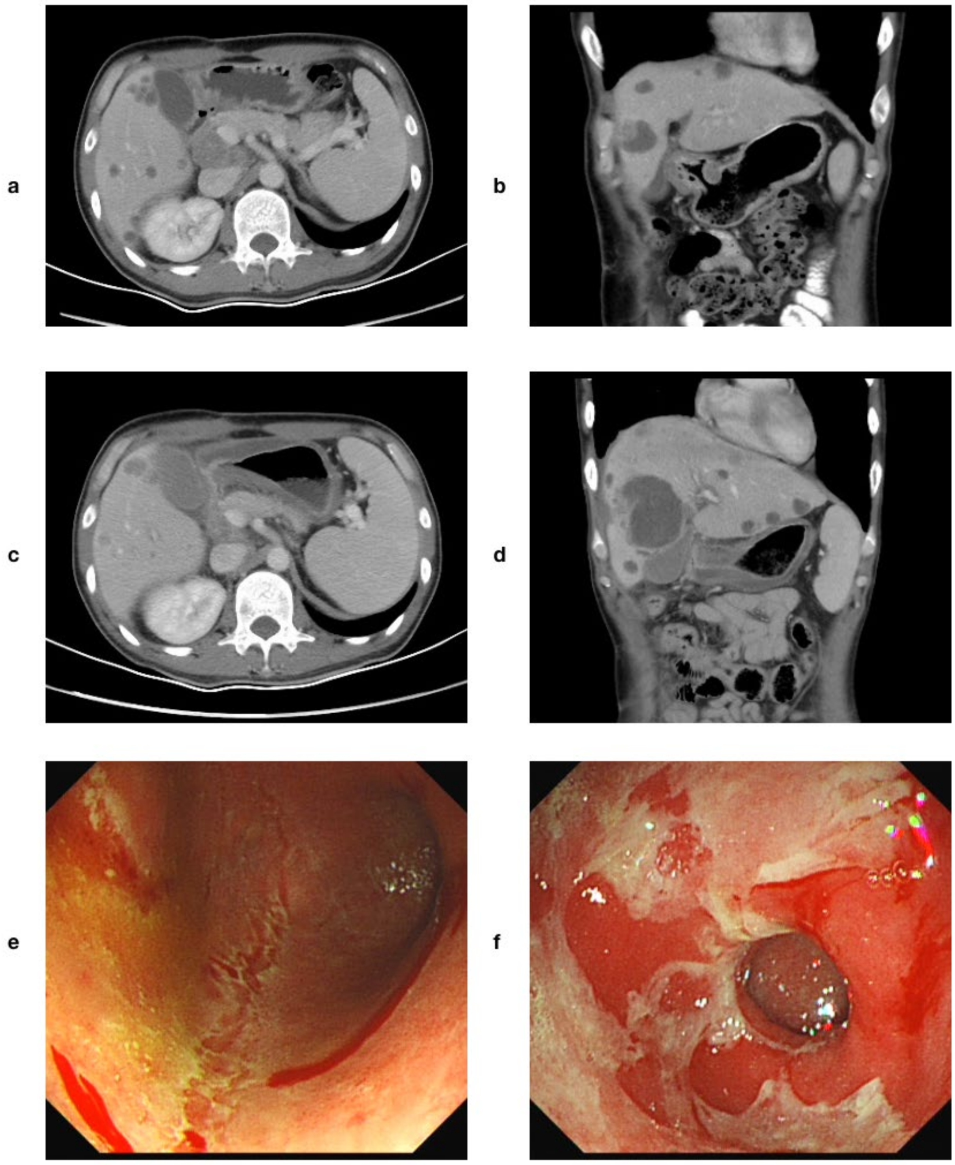

Fig. S3 | Gastric Mucosal Edema and Erosion After CT041 Infusion
The images show edema and erosion of the gastric mucosa after CT041 infusion for Pt17, a 34-year-old male with neuroendocrine carcinoma of the rectum. **a and b** show CT images of his stomach status before CT041 infusion. **c and d** show CT images of the diffused edema of gastric mucosa after CT041 infusion; **e and f** show gastroscopy photographs of the diffused erosion of gastric mucosa.

Pt 01

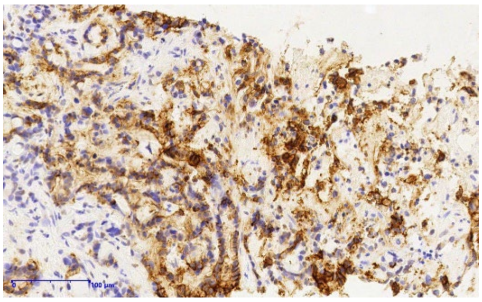

Pre-infusion 2+ 50%

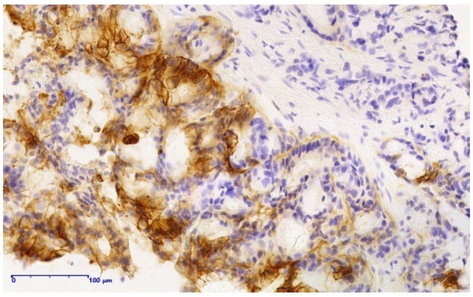

Post-infusion 3+ 50%

Pt 06

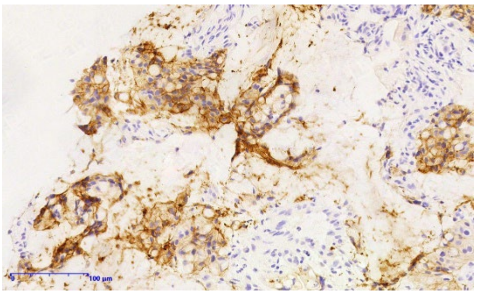

Pre-infusion 2+ 60%

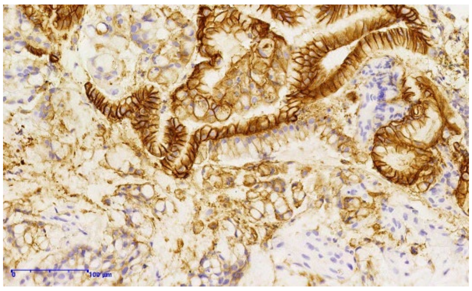

Post-infusion 2+ 50%

Pt 07

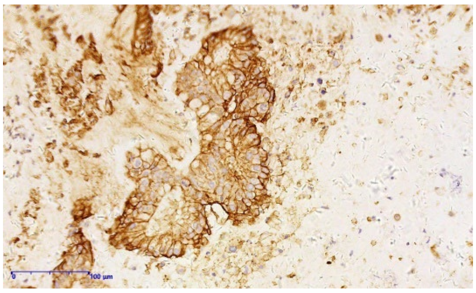

Pre-infusion 3+ 80%

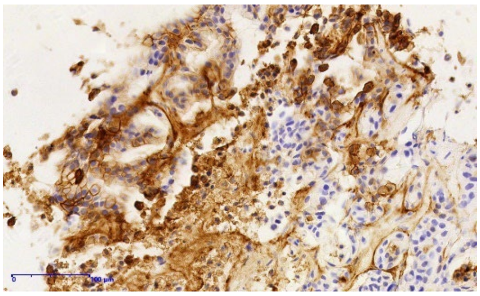

Post-infusion 3+ 90%

Fig. S4 | Expression of CLDN18.2 Pre and Post CT041 Infusion

CLDN18.2 expression of Pt 01, Pt 06 and Pt 07 after the first infusion and before the second infusion (week 4 after first CT041 infusion for Pt 01, week 16 after first CT041 infusion for Pt 06 and week 6 after first CT041 infusion for Pt 07).
The expression of CLDN18.2 was detected by immunohistochemistry.

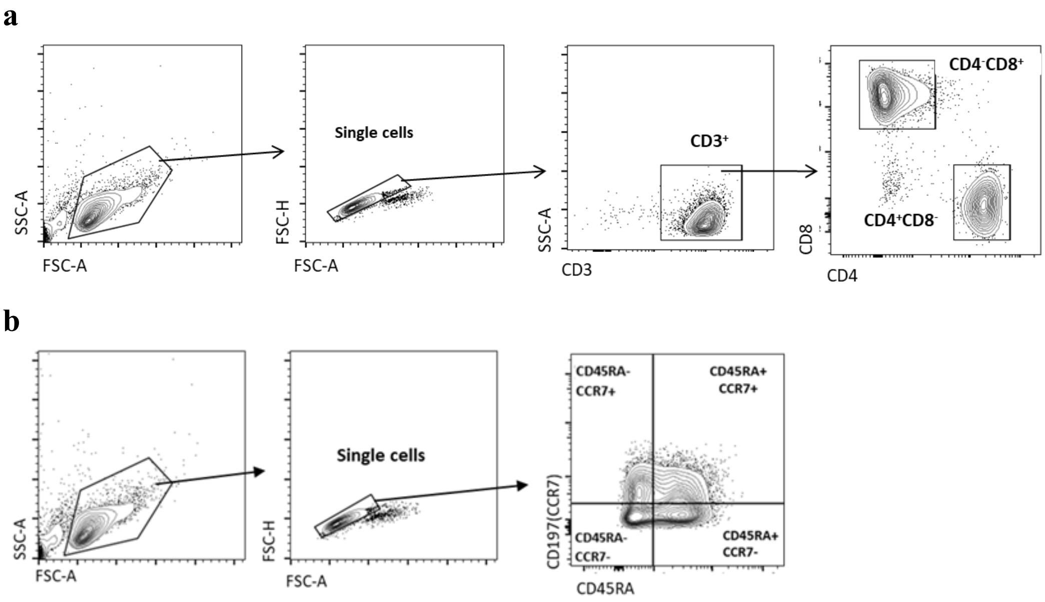

Fig. S5 | Gating strategies in phenotyping analysis

a, Gating strategy to determine the percentage of $CD3^+CD4^+CD8^-$ (Th) cells and $CD3^+CD4^-CD8^+$ (cytotoxic T lymphocyte, CTL) cells.

b, Gating strategy to determine the percentage of $CD45RA^+$ /$CCR7^+$ (naive T) cells, $CD45RA^-$ /$CCR7^+$ (central memory T) cells, $CD45RA^-$/$CCR7^-$ (effector memory T) cells, and $CD45RA^+$ /$CCR7^-$ (terminally differentiated effector T) cells in CT041 products.

Table S1 | Summary of Survival Follow-up (mITT)

	Dose Escalation/De-escalation			Dose Expansion	All
	2.5×10^8 (n=6)	3.75×10^8 (n=6)	5×10^8 (n=3)	2.5×10^8 (n=22)	Total (N=37)
**Reverse KM survival follow-up time***					
Median (95% CI)	10.9 [10.2, NE]	6.7 [6.7, NE]	19.4 [NE, NE]	8.3 [4.4, 8.8]	8.5 [7.4, 9.3]
Min, max	3.6, 17.7	2.9, 7.4	4.9, 19.4	2.9, 10	2.9, 19.4

*Reverse Kaplan-Meier estimate of survival follow-up time based on leukapheresis date.
Survival follow-up time based on leukapheresis date (months) = (Death date or cutoff date - leukapheresis date) / 30.4375
Modified intention-to-treat analysis set (mITT): Includes all subjects who received CT041 infusion in the study.

Table S2| Intervals between CT041 Infusions (mITT)

**Total number of CT041 treatment cycles**	**Patients, n (%) (N=37)**
1	19 (51.4)
2	15 (40.5)
3	3 (8.1)
**Intervals between CT041 infusions**	**Duration (days)**
Apheresis to first CT041 infusion	
Median (days)	27
Min, max (days)	22, 187
First to second CT041 infusion	
Median (days)	72
Min, Max (days)	35, 211
Second to third CT041 infusion	
Median (days)	101
Min, max (days)	81, 140

Table S3 | Summary of the Severity of Treatment-Related AEs Based on SOC and PT (≥10%, grouped by phase), any Grade 3 or 4 Treatment-Related AE, and any nervous system disorders (Safety Analysis Set)*

System Organ Class<br>Preferred Term, n (%)	Dose Escalation/De-escalation						Dose Expansion		All	
	CT041 2.5×10^8 (n=6)		CT041 3.75×10^8 (n=6)		CT041 5.0×10^8 (n=3)		CT041 2.5×10^8 (n=22)		Total (N=37)	
	G 3/4	Any	G 3/4	Any	G 3/4	Any	G 3/4	Any	G 3/4	Any
**Any treatment related adverse event**	6 (100)	6 (100)	6 (100)	6 (100)	3 (100)	3 (100)	22 (100)	22 (100)	37 (100)	37 (100)
**Investigations**	6 (100)	6 (100)	6 (100)	6 (100)	3 (100)	3 (100)	22 (100)	22 (100)	37 (100)	37 (100)
Hemoglobin decreased	0	6 (100)	3 (50.0)	6 (100)	2 (66.7)	3 (100)	10 (45.5)	22 (100)	15 (40.5)	37 (100)
Lymphocyte count decreased	6 (100)	6 (100)	6 (100)	6 (100)	3 (100)	3 (100)	22 (100)	22 (100)	37 (100)	37 (100)
White blood cell count decreased	4 (66.7)	6 (100)	6 (100)	6 (100)	3 (100)	3 (100)	18 (81.8)	21 (95.5)	31 (83.8)	36 (97.3)
Neutrophil count decreased	4 (66.7)	4 (66.7)	4 (66.7)	6 (100)	3 (100)	3 (100)	14 (63.6)	20 (90.9)	25 (67.6)	33 (89.2)
Protein total decreased	0	6 (100)	0	6 (100)	0	3 (100)	0	18 (81.8)	0	33 (89.2)
Blood albumin decreased	0	5 (83.3)	0	4 (66.7)	0	3 (100)	0	16 (72.7)	0	28 (75.7)
Occult blood positive	0	3 (50.0)	0	4 (66.7)	0	3 (100)	0	15 (68.2)	0	25 (67.6)
Platelet count decreased	1 (16.7)	4 (66.7)	1 (16.7)	4 (66.7)	1 (33.3)	3 (100)	3 (13.6)	11 (50.0)	6 (16.2)	22 (59.5)
Activated partial thromboplastin time prolonged	0	1 (16.7)	0	6 (100)	0	0	0	12 (54.5)	0	19 (51.4)
Aspartate aminotransferase increased	0	3 (50.0)	1 (16.7)	3 (50.0)	0	2 (66.7)	2 (9.1)	11 (50.0)	3 (8.1)	19 (51.4)
Prothrombin time prolonged	0	1 (16.7)	0	4 (66.7)	0	2 (66.7)	0	12 (54.5)	0	19 (51.4)
Alanine aminotransferase increased	0	3 (50.0)	2 (33.3)	4 (66.7)	0	2 (66.7)	0	9 (40.9)	2 (5.4)	18 (48.6)
Bilirubin conjugated increased	0	1 (16.7)	1 (16.7)	5 (83.3)	0	0	4 (18.2)	11 (50.0)	5 (13.5)	17 (45.9)
Blood fibrinogen decreased	1 (16.7)	4 (66.7)	1 (16.7)	1 (16.7)	0	1 (33.3)	0	8 (36.4)	2 (5.4)	14 (37.8)
Lipase increased	0	0	0	2 (33.3)	0	0	3 (13.6)	10 (45.5)	3 (8.1)	12 (32.4)
Blood bilirubin increased	0	0	1 (16.7)	3 (50.0)	0	0	0	6 (27.3)	1 (2.7)	9 (24.3)
Protein urine present	0	1 (16.7)	0	1 (16.7)	0	0	0	3 (13.6)	0	5 (13.5)
Prothrombin level decreased	0	0	0	0	0	0	0	5 (22.7)	0	5 (13.5)
Oxygen saturation decreased	0	1 (16.7)	0	0	0	0	0	3 (13.6)	0	4 (10.8)
Prothrombin time ratio increased	0	0	0	0	0	0	0	4 (18.2)	0	4 (10.8)
Prothrombin time shortened	0	0	0	2 (33.3)	0	0	0	2 (9.1)	0	4 (10.8)
Amylase increased	0	0	0	0	0	0	1 (4.5)	3 (13.6)	1 (2.7)	3 (8.1)
**General disorders and administration site conditions**	1 (16.7)	5 (83.3)	0	6 (100)	1 (33.3)	3 (100)	1 (4.5)	22 (100)	3 (8.1)	36 (97.3)
Pyrexia	1 (16.7)	5 (83.3)	0	6 (100)	1 (33.3)	3 (100)	1 (4.5)	22 (100)	3 (8.1)	36 (97.3)
Oedema peripheral	0	2 (33.3)	0	1 (16.7)	0	2 (66.7)	0	5 (22.7)	0	10 (27.0)
**Immune system disorders**	0	5 (83.3)	0	6 (100)	1 (33.3)	3 (100)	0	21 (95.5)	1 (2.7)	35 (94.6)
Cytokine release syndrome	0	5 (83.3)	0	6 (100)	0	3 (100)	0	21 (95.5)	0	35 (94.6)
Anaphylactic shock	0	0	0	0	1 (33.3)	1 (33.3)	0	0	1 (2.7)	1 (2.7)
**Gastrointestinal disorders**	0	3 (50.0)	0	6 (100)	1 (33.3)	3 (100)	1 (4.5)	19 (86.4)	2 (5.4)	31 (83.8)
Nausea	0	2 (33.3)	0	4 (66.7)	0	1 (33.3)	0	13 (59.1)	0	20 (54.1)
Vomiting	0	2 (33.3)	0	1 (16.7)	0	1 (33.3)	0	10 (45.5)	0	14 (37.8)
Diarrhea	0	2 (33.3)	0	2 (33.3)	0	1 (33.3)	0	5 (22.7)	0	10 (27.0)
Abdominal distension	0	1 (16.7)	0	2 (33.3)	0	0	0	4 (18.2)	0	7 (18.9)

System Organ Class<br>Preferred Term, n (%)	Dose Escalation/De-escalation						Dose Expansion		All	
	CT041 2.5×10^8 (n=6)		CT041 3.75×10^8 (n=6)		CT041 5.0×10^8 (n=3)		CT041 2.5×10^8 (n=22)		Total (N=37)	
	G 3/4	Any	G 3/4	Any	G 3/4	Any	G 3/4	Any	G 3/4	Any
Abdominal pain	0	3 (50.0)	0	1 (16.7)	0	0	0	3 (13.6)	0	7 (18.9)
Abdominal pain upper	0	0	0	1 (16.7)	0	2 (66.7)	0	2 (9.1)	0	5 (13.5)
Gastritis	0	0	0	0	0	0	0	4 (18.2)	0	4 (10.8)
Gastroesophageal reflux disease	0	0	0	0	0	2 (66.7)	0	2 (9.1)	0	4 (10.8)
Gastrointestinal hemorrhage	0	0	0	0	1 (33.3)	1 (33.3)	0	1 (4.5)	1 (2.7)	2 (5.4)
Gastritis erosive	0	0	0	0	0	0	1 (4.5)	1 (4.5)	1 (2.7)	1 (2.7)
**Metabolism and nutrition disorders**	1 (16.7)	3 (50.0)	1 (16.7)	5 (83.3)	0	1 (33.3)	0	14 (63.6)	2 (5.4)	23 (62.2)
Decreased appetite	0	0	0	3 (50.0)	0	1 (33.3)	0	8 (36.4)	0	12 (32.4)
Hyponatremia	1 (16.7)	2 (33.3)	1 (16.7)	3 (50.0)	0	0	0	6 (27.3)	2 (5.4)	11 (29.7)
Hypokalemia	0	1 (16.7)	0	3 (50.0)	0	0	0	6 (27.3)	0	10 (27.0)
**Skin and subcutaneous tissue disorders**	1 (16.7)	4 (66.7)	0	3 (50.0)	1 (33.3)	2 (66.7)	0	5 (22.7)	2 (5.4)	14 (37.8)
Rash	1 (16.7)	2 (33.3)	0	3 (50.0)	1 (33.3)	2 (66.7)	0	5 (22.7)	2 (5.4)	12 (32.4)
Pruritus	0	0	0	1 (16.7)	0	0	0	3 (13.6)	0	4 (10.8)
**Cardiac disorders**	0	1 (16.7)	0	0	0	2 (66.7)	0	9 (40.9)	0	12 (32.4)
Sinus tachycardia	0	0	0	0	0	1 (33.3)	0	4 (18.2)	0	5 (13.5)
Tachycardia	0	0	0	0	0	0	0	5 (22.7)	0	5 (13.5)
**Respiratory, thoracic and mediastinal disorders**	0	3 (50.0)	0	1 (16.7)	0	3 (100)	0	5 (22.7)	0	12 (32.4)
Chest pain	0	1 (16.7)	0	0	0	2 (66.7)	0	2 (9.1)	0	5 (13.5)
Dyspnea	0	1 (16.7)	0	0	0	2 (66.7)	0	2 (9.1)	0	5 (13.5)
Pleural effusion	0	2 (33.3)	0	0	0	2 (66.7)	0	0	0	4 (10.8)
**Vascular disorders**	0	1 (16.7)	0	0	0	2 (66.7)	0	4 (18.2)	0	7 (18.9)
Hypotension	0	1 (16.7)	0	0	0	2 (66.7)	0	3 (13.6)	0	6 (16.2)
**Nervous system disorders**	0	1 (16.7)	0	0	0	1 (33.3)	0	3 (13.6)	0	5 (13.5)
Dizziness	0	0	0	0	0	0	0	2 (9.1)	0	2 (5.4)
Headache	0	1 (16.7)	0	0	0	0	0	0	0	1 (2.7)
Hypoesthesia	0	0	0	0	0	0	0	1 (4.5)	0	1 (2.7)
Somnolence	0	0	0	0	0	1 (33.3)	0	0	0	1 (2.7)
**Injury, poisoning and procedural complications**	0	0	0	0	1 (33.3)	1 (33.3)	0	0	1 (2.7)	1 (2.7)
Anastomotic fistula	0	0	0	0	1 (33.3)	1 (33.3)	0	0	1 (2.7)	1 (2.7)

*CRS and ICANS were graded according to ASTCT 2019 criteria. No ICANS or psychiatric toxicities were observed in the study. The other AEs were coded according to Medical Dictionary for Regulatory Activities (version 23.1), graded according to Common Terminology Criteria for Adverse Events version 5.0.

Treatment-related adverse events include AEs related to preconditioning or CT041.

Table S4 | Summary of Time to Recovery for Hematological Toxicity with Grade≥3 Occurred within D28 After First Infusion (Safety Analysis Set)

Variable	Dose Escalation/De-escalation			Dose Expansion	All
	2.5×10^8 cells (n=6)	3.75×10^8 cells (n=6)	5×10^8 cells (n=3)	2.5×10^8 cells (n=22)	Total (N=37)
**Patients with white blood cell count decreased (leukopenia) Grade ≥ 3, n (%)**	4 (66.7)	6 (100)	3 (100)	18 (81.8)	31 (83.8)
Recovered to ≤ Grade 2, n (%)	4 (100)	6 (100)	3 (100)	18 (100)	31 (100)
Recovery time, median (min, max)	3 (1, 8)	8 (4, 12)	3 (2, 5)	7.5 (2, 18)	6 (1, 18)
Without recovery at day 28 after CT041 infusion, n (%)	0	0	0	2 (11.1)	2 (6.5)
**Patients with neutrophil count decreased (neutropenia) Grade ≥ 3, n (%)**	3 (50.0)	3 (50.0)	3 (100)	13 (59.1)	22 (59.5)
Recovered to ≤ Grade 2, n (%)	3 (100)	3 (100)	3 (100)	13 (100)	22 (100)
Recovery time, median (min, max)	9 (4, 11)	4 (3, 27)	5 (2, 7)	6 (2, 36)	5.5 (2, 36)
Without recovery at day 28 after CT041 infusion, n (%)	0	1 (33.3)	0	1 (7.7)	2 (9.1)
**Patients with platelet count decreased (thrombocytopenia) Grade ≥ 3, n (%)**	1 (16.7)	1 (16.7)	0	3 (13.6)	5 (13.5)
Recovered to ≤ Grade 2, n (%)	1 (100)	1 (100)	0	3 (100)	5 (100)
Recovery time, median (min, max)	4 (4, 4)	106 (106, 106)	0	4 (1, 11)	4 (1, 106)
Without recovery at day 28 after CT041 infusion, n (%)	0	1 (100)	0	0	1 (20.0)
**Patients with hemoglobin Decreased (anemia) Grade ≥ 3, n (%)**	0	2 (33.3)	1 (33.3)	8 (36.4)	11 (29.7)
Recovered to ≤ Grade 2, n (%)	0	2 (100)	1 (100)	6 (75.0)	9 (81.8)
Recovery time Kaplan-Meier estimated, median (95% CI)					
Median	0	53	4	12.5 [4.0, NE]	9.0 [4.0, NE]
Without recovery at day 28 after CT041 infusion, n (%)	0	1 (50.0)	1 (100)	4 (50.0)	6 (54.5)

NE, not evaluable.

Table S5 | Summary of Cytokine Release Syndrome Incidence and Treatment (Safety Analysis Set)

After First CT041 Infusion	$2.5×10^8$ (n=28)	$3.75×10^8$ (n=6)	$5×10^8$ (n=3)	Total (N=37)
**Number of patients with CRS, n (%)**	26 (92.9)	6 (100)	3 (100)	35 (94.6)
Grade 1, n (%)	14 (50)	4 (66.7)	0	18 (48.6)
Grade 2, n (%)	12 (42.9)	2 (33.3)	3 (100)	17 (45.9)
**CRS onset day (days after first infusion)**				
Median (Min, max)	2 (1, 3)	2 (1, 3)	2 (1, 2)	2 (1, 3)
**CRS duration (days)**				
Median (Min, max)	5.5 (3, 29)	6 (3, 17)	22 (22, 42)	6 (3, 42)
**Glucocorticoids, n (%)**	2 (7.1)	0	1 (33.3)	3 (8.1)
**Dexamethasone sodium phosphate, n (%)**	1 (3.6)	0	1 (33.3)	2 (5.4)
**Average daily dose(mg)**				
Median (Min, max)	5 (5, 5)	-	10 (10, 10)	7.5 (5, 10)
**Methylprednisolone sodium succinate, n (%)**	1 (3.6)	0	0	1 (2.7)
**Average daily dose (mg)**				
Median (Min, max)	40 (40, 40)	-	-	40 (40, 40)
**Tocilizumab, n (%)**	22 (78.6)	2 (33.3)	3 (100)	27 (73)
**Average daily dose (mg)**				
Median (Min, max)	320 (160, 480)	280 (240, 320)	480 (432, 513)	320 (160, 513)
**Average daily dose by weight (mg)**				
Median (Min, max)	6.15 (4.3, 8.3)	6.14 (6, 6.3)	7.89 (7.7, 8.5)	6.15 (4.3, 8.5)
**After Second CT041 Infusion**	**$2.5×10^8$ (n=28)**	**$3.75×10^8$ (n=6)**	**$5×10^8$ (n=3)**	**Total (N=37)**
**Number of patients with CRS, n (%)**	9 (64.3)	1 (100)	3 (100)	13 (72.2)
Grade 1, n (%)	6 (42.9)	1 (100)	1 (33.3)	8 (44.4)
Grade 2, n (%)	3 (21.4)	0	2 (66.7)	5 (27.8)
**CRS onset day (days after second infusion)**				
Median (Min, max)	2 (1, 4)	3 (3, 3)	2 (1, 8)	2 (1, 8)
**CRS duration (days)**				
Median (Min, max)	3 (2, 7)	6 (6, 6)	13 (3, 30)	4 (2, 30)
**Glucocorticoids, n(%)**	0	0	1 (33.3)	1 (5.6)
**Methylprednisolone sodium succinate, n(%)**	0	0	1 (33.3)	1 (5.6)
**Average daily dose (mg)**				
Median (Min, max)	-	-	61.54 (61.5, 61.5)	61.54 (61.5, 61.5)

Table S6 | Gastric Mucosal Injuries (Safety Analysis Set)

Pt. No.	CT041 dose (cells)	Age/sex	Peak CAR copies (copies/µg gDNA)	CLDN18.2 expression	AE term/ Preferred term	Start date/ Days after first CT041 infusion	End date/ Days after first CT041 infusion	Onset cycle/ Days after first CT041 infusion	AE duration (days)	CTCAE Grade /Outcome	Relationship to CT041	Caused discontinuation from the study?
Pt 11	2.5×10^{8}	30/F	19495	++, 20%	Erosion of gastric mucosa/ Gastritis erosive	2020-08-18/ 43	2020-09-16/ 72	1/43	30	Grade 3/ Recovered	Possibly Related	No
Pt 16	2.5×10^{8}	63/M	7339	++, 10%	Residual gastritis/ Gastritis	2020-08-26/ 16	2020-09-04/ 25	1/16	10	Grade 2/ Recovered	Possibly Related	No
Pt 23	2.5×10^{8}	39/M	24503	+++, 90%	Congestive gastritis/ Gastritis	2020-09-14/ 20	2020-12-02/ 99	1/20	80	Grade 2/ Recovered	Possibly Related	No
Pt 24	2.5×10^{8}	73/M	10849	++, 40%	All gastritis/ Gastritis	2020-09-11/ 10	2020-09-29/ 28	1/10	19	Grade 2/ Recovered	Possibly Related	No
Pt 17	2.5×10^{8}	34/M	119581	+++, 60%	All gastritis/ Gastritis	2020-09-14/ 31	Ongoing	1/31	NA	Grade 1/ Not Recovered	Related	No
Pt 35	2.5×10^{8}	39/F	2408	+++, 90%	Gastric mucosal lesion/ Gastric mucosal lesion	2021-01-14/ 17	2021-01-25/ 28	1/17	12	Grade 2/ Recovered	Possibly Related	No

Table S7 | Efficacy Evaluation Based on Investigator Assessment Per RECIST 1.1

Variable	Gastric cancer patients (n=28)	Failed 2 prior lines, gastric cancer patients (n=24)	Failed to Prior 2 lines, gastric cancer patients 2.5×10^8 (n=18)	Other digestive system cancers (n=9)	All patients (N=37)
**Best overall response***					
Complete response (CR), n (%)	0	0	0		0
Partial response (PR), n (%)	16 (57.1)	13 (54.2)	11 (61.1)	2	18 (48.6)
Stable disease (SD), n (%)	5 (17.9)	5 (20.8)	4 (22.2)	4	9 (24.3)
Progressive disease (PD), n (%)	7 (25.0)	6 (25.0)	3 (16.7)	3	10 (27.0)
Indeterminate response (NE), n (%)	0	0	0	0	0
**Objective response rate (ORR), n (%)**	16(57.1)	13(54.2)	11(61.1)	2(22.2)	18(48.6)
**[95% CI]**	[37.18, 75.54]	[32.82, 74.45]	[35.75, 82.70]	[2.81, 60.01]	[31.92, 65.60]
**Disease control rate (DCR), n (%)**	21(75.0)	18(75)	15(83.3)	6(66.7)	27(73.0)
**[95% CI]**	[55.13, 89.31]	[53.29, 90.23]	[58.58, 96.42]	[29.93, 92.51]	[55.88, 86.21]
**mPFS (months)**	4.2 [3.7, 9.2]	5.4 [2.6, 9.2]	5.6 [2.6, 9.2]	2.6 [1.8, 3.5]	3.7 [2.6, 5.4]
**OS rate at 6 months (%)**	81.2 [60.3, 91.8]	83.3 [61.5, 93.4]	83.3 [56.8, 94.3]	77.8 (36.5, 93.9)	80.1 [62.5, 90.0]
**DOR rate at 6 months (%)**	53.3 [20.7, 77.8]	53.5 [17.2, 80.0]	57.1 [17.2, 83.7]	NA	44.8 [17.3, 69.3]

* Response rate was confirmed at least 4 weeks post the first evaluation.

Table S8 | Summary of Anti-drug Antibody (ADA) Results

	D0	W4	W8	W12	W18	W24	W32	W40	W48	W56	Overall
No. with available ADA results	37	37	26	21	15	7	4	1	1	1	37
No. (%) ADA-positive	2 (5.4)	18 (48.6)	11 (42.3)	13 (61.9)	10 (66.7)	6 (85.7)	4 (100)	1 (100)	1 (100)	1 (100)	28 (75.7)
Titer, median (Q1, Q3)	1.0 (1.0, 1.0)	2.0 (1.0, 16.0)	1.0 (1.0, 5.0)	9.0 (3.0, 54.0)	8.0 (2.0, 56.0)	62.0 (6.0, 101.0)	535.5 (263.5, 556.0)	705.0 (705.0, 705.0)	1467.0 (1142.0, 1792.0)	2716.0 (2716.0, 2716.0)	8.0 (1.0, 78.0)
Titer, (min, max)	1, 1	1, 2454	1, 3892	1, 465	1, 278	1, 115	6, 562	705, 705	1142, 1792	2716, 2716	1, 3892

Table S9 | Reagent and Sequence for qPCR analysis of CAR CLDN18.2 cell expansion and persistence

qPCR Reagent	Sequence
CAR-CLDN18.2 FAM Probe	5'-CTGAGCAGCGTGACCGCCGC-3'
Forward Primer CAR-CLDN18.2	5'-TGGAGTGGATCGGCTACATC-3'
Reverse Primer CAR-CLDN18.2	5'-AGTAGTAGATGGCGGTGTCG-3'
