# Supplementary material for: Claudin18.2-specific CAR T cells in gastrointestinal cancers: phase 1 trial interim results
Source: Nat Med. 2022 May 9;28(6):1189–98. doi: 10.1038/s41591-022-01800-8 (PMC9205778; doi:10.1038/s41591-022-01800-8)
Supplement: Supplementary file 2 — Reporting Summary [file 41591_2022_1800_MOESM2_ESM.pdf]

nature portfolio

Corresponding author(s): Lin Shen

Last updated by author(s): Mar 18, 2022

Reporting Summary

Nature Portfolio wishes to improve the reproducibility of the work that we publish. This form provides structure for consistency and transparency in reporting. For further information on Nature Portfolio policies, see our Editorial Policies and the Editorial Policy Checklist.

Statistics

For all statistical analyses, confirm that the following items are present in the figure legend, table legend, main text, or Methods section.

n/a	Confirmed	
☐	☒	The exact sample size (*n*) for each experimental group/condition, given as a discrete number and unit of measurement
☐	☒	A statement on whether measurements were taken from distinct samples or whether the same sample was measured repeatedly
☐	☒	The statistical test(s) used AND whether they are one- or two-sided<br>*Only common tests should be described solely by name; describe more complex techniques in the Methods section.*
☐	☒	A description of all covariates tested
☒	☐	A description of any assumptions or corrections, such as tests of normality and adjustment for multiple comparisons
☐	☒	A full description of the statistical parameters including central tendency (e.g. means) or other basic estimates (e.g. regression coefficient) AND variation (e.g. standard deviation) or associated estimates of uncertainty (e.g. confidence intervals)
☐	☒	For null hypothesis testing, the test statistic (e.g. *F*, *t*, *r*) with confidence intervals, effect sizes, degrees of freedom and *P* value noted<br>*Give P values as exact values whenever suitable.*
☒	☐	For Bayesian analysis, information on the choice of priors and Markov chain Monte Carlo settings
☒	☐	For hierarchical and complex designs, identification of the appropriate level for tests and full reporting of outcomes
☐	☒	Estimates of effect sizes (e.g. Cohen's *d*, Pearson's *r*), indicating how they were calculated

Our web collection on statistics for biologists contains articles on many of the points above.

Software and code

Policy information about availability of computer code

Data collection	Taimei eCollect System， Version 5.11
Data analysis	SAS, Version 9.4

For manuscripts utilizing custom algorithms or software that are central to the research but not yet described in published literature, software must be made available to editors and reviewers. We strongly encourage code deposition in a community repository (e.g. GitHub). See the Nature Portfolio guidelines for submitting code & software for further information.

Data

Policy information about availability of data

All manuscripts must include a data availability statement. This statement should provide the following information, where applicable:

- Accession codes, unique identifiers, or web links for publicly available datasets
- A description of any restrictions on data availability
- For clinical datasets or third party data, please ensure that the statement adheres to our policy

All data used in the interim analyses supporting the findings of the present study are available within the manuscript and its supplementary information files. All requests for further data sharing will be reviewed by the leading clinical center, Department of Gastrointestinal Oncology, Peking University Cancer Hospital and Institute, and the study collaborator, CARsgen Therapeutics Co., Ltd, to verify whether the request is subject to any intellectual property or confidentiality obligations. Figure 1a-1c, Figure 2a-2c, Extended Data Table 1, Extended Data Fig. 3-4, 6-7 present individual participant-level data with privacy information de-identified to support understanding of the study results. Further requests for access to the individual participant level data from this study can be submitted via email to the corresponding author with detailed proposals. Each participant’s rights and privacy are key subjects to take into consideration while sharing information. A signed data access agreement with the collaborator is required before accessing shared data.

Field-specific reporting

Please select the one below that is the best fit for your research. If you are not sure, read the appropriate sections before making your selection.

☒ Life sciences ☐ Behavioural & social sciences ☐ Ecological, evolutionary & environmental sciences

For a reference copy of the document with all sections, see nature.com/documents/nr-reporting-summary-flat.pdf

Life sciences study design

All studies must disclose on these points even when the disclosure is negative.

Sample size	The sample size was based on clinical considerations and a typical "3+3" dose-escalation/de-escalation design.
Data exclusions	Data from participants infused CT041 and completed at least 12 weeks visit were included. 2 participants did not infused CT041 after lymphodepletion and 12 participants infused CT041 but has not completed 12 weeks follow-up were not included in this interim analysis.
Replication	Replication is not applicable and not planned as this is a phase 1 study in the early stage of clinical development for dose-selection and dose-expansion.
Randomization	It is a single arm study.
Blinding	It is an open-lable study.

Reporting for specific materials, systems and methods

We require information from authors about some types of materials, experimental systems and methods used in many studies. Here, indicate whether each material, system or method listed is relevant to your study. If you are not sure if a list item applies to your research, read the appropriate section before selecting a response.

Materials & experimental systems

n/a	Involved in the study	
☐	☒	Antibodies
☒	☐	Eukaryotic cell lines
☒	☐	Palaeontology and archaeology
☒	☐	Animals and other organisms
☐	☒	Human research participants
☐	☒	Clinical data
☒	☐	Dual use research of concern

Methods

n/a	Involved in the study	
☒	☐	ChIP-seq
☐	☒	Flow cytometry
☒	☐	MRI-based neuroimaging

Antibodies

Antibodies used	Mouse Anti-CLDN18.2 monoclonal antibody<br>Goat anti hu8E5-2I scFv polyclonal antibody<br>BD Tritest CD4 FITC/CD8 PE/CD3; PerCP BD; 340298; CD3-SK7/CD4-SK3/CD8-SK1; 82786/90711/33593/51929<br>BV421 Mouse Anti-HumanCD197 (CCR7); BD; 562555, 150503 ; 8291773/9304147/9224909<br>FITC Mouse Anti-Human CD45RA; BD; 555488, HI100; 8037739/9107502<br>Each antibody was used in an undiluted form.
Validation	All the antibodies panels was validated separately in the series of the analytical validation runs of the CLDN18.2 immunohistochemistry assay, the flow cytometry and the ADA assay prior to use in this clinical trial.

Human research participants

Policy information about studies involving human research participants

Population characteristics	Male or female patients aged between 18 and 75 years were eligible if they had histologically confirmed, previously treated, advanced digestive system cancers. CLDN18.2 expression were histologically confirmed positive with tumor tissues. Patients with the active infection including but not limited to HBV, HCV, tuberculosis, brain metastases, unstable or active gastric ulcer, or gastroenterological bleeding were excluded.
Recruitment	Eligible participants were recruited at the discretion of the investigator. When needed, participants eligibility were discussed with collaborator. Patients were enrolled based on disease characteristics, suitability and eligibility for the trial. It is not considered there was any selection bias in recruitment of patients into the trial.

Ethics oversight | Protocols were approved by IEC of Department of Gastrointestinal Oncology, Peking University Cancer Hospital

Note that full information on the approval of the study protocol must also be provided in the manuscript.

Clinical data

Policy information about clinical studies

All manuscripts should comply with the ICMJE guidelines for publication of clinical research and a completed CONSORT checklist must be included with all submissions.

Clinical trial registration: NCT03874897

Study protocol: The clinical protocol could not be included because it contains proprietary information

Data collection: Participants' data were entered to electronic case report form by investigator or authorized site staff at study sites. The first screening consent form was signed on 16 April 2019 and the data collection started then. The study recruitment and data collection are continuous and cutoff data for this interim analyses was 8 April 2021.

Outcomes: Commonly used safety and efficacy clinical endpoints for oncology clinical trial were selected. Safety and efficacy assessment were performed by investigators according to CTCAE, ASTCT or RECIST 1.1 and analyzed per SAP. The primary objective was to evaluate the safety and tolerability in 4 weeks(28 days) post-infusion. The secondary endpoints were the preliminary efficacy in patients infused CT041, pharmaceutics of CT041 AND the correlations between T-cell subset frequencies or anti-CAR T antibody and clinical activities.

Flow Cytometry

Plots

Confirm that:

☒ The axis labels state the marker and fluorochrome used (e.g. CD4-FITC).

☒ The axis scales are clearly visible. Include numbers along axes only for bottom left plot of group (a 'group' is an analysis of identical markers).

☒ All plots are contour plots with outliers or pseudocolor plots.

☒ A numerical value for number of cells or percentage (with statistics) is provided.

Methodology

Sample preparation: Samples for flow cytometry were from CAR T cells for formulation.

Instrument: Samples (3.0×10^5 cells) were stained and analyzed

Software: FlowJo™ v10.5 Software (Tree Star Inc., Ashland, OR, USA)

Cell population abundance: the FACS Canto II (BD Biosciences) flow cytometer

Gating strategy: Gating for positive staining was determined according to the separation of negative and positive cells or fluorescence minus one (FMO) control when necessary. The result of cell phenotype is expressed as the percentage of positive cells.

☒ Tick this box to confirm that a figure exemplifying the gating strategy is provided in the Supplementary Information.
